# Scalable nanoconfined ionic liquid membranes with ultrapermeance and ultraselectivity for efficient CO_2_ capture

**DOI:** 10.1126/sciadv.aea1329

**Published:** 2026-01-07

**Authors:** Fan Wang, Dinesh Kumar Behera, Bratin Sengupta, David Li, Miao Yu

**Affiliations:** ^1^ Department of Chemical and Biological Engineering, University at Buffalo, Buffalo, NY 14260, USA.; ^2^ Stevenson High School, 1 Stevenson Dr, Lincolnshire, IL 60069, USA.; ^3^ RENEW Institute, University at Buffalo, Buffalo, NY 14260, USA.

## Abstract

Supported ionic liquid membranes (SILMs) stand attractive for gas separation considering the tunability, high gas selectivity, and ease of fabrication. However, the intrinsic instability of SILMs limited their practical use. Here, we designed, fabricated, and investigated an ultrapermeable, ultraselective, and stable nanoconfined ionic liquid (NCIL) membrane for highly efficient CO_2_ capture. Specifically, the combination of a thin, open, and uniform nanoconfined network of single-walled carbon nanotube with highly CO_2_–selective ionic liquid carrier enabled the superior CO_2_ permeance of 1654 GPU and CO_2_/N_2_ selectivity of 1132, surpassing most state-of-the-art facilitated transport membranes. The scale-up potential of the NCIL membrane was demonstrated under simulated natural gas flue gas conditions, achieving CO_2_ enrichment from 4.2 to 98% in a single step. Given the processability and scalability of NCIL membrane, this work affirms the industrial potential of SILMs and offers a viable strategy for designing and fabricating stable SILMs for gas separation.

## Teaser

Scalable nanoconfined ionic liquid membranes enable one-step CO_2_ enrichment to 98%.

## INTRODUCTION

Excessive CO_2_ emissions, driven by rapidly increasing human activity since the early 1900s, have intensified the greenhouse effect (*1*), leading to severe climate impacts such as more frequent extreme weather events, rising sea levels (*2*), and ocean acidification. Capturing CO_2_ from point sources is widely recognized as critical for mitigating climate change (*3*, *4*). A large portion of point-source CO_2_ emissions originates from the postcombustion or precombustion of fossil fuels in electricity generation (*5*, *6*). As of 2025, natural gas combustion in natural gas combined cycle power plants accounted for 43% of electricity generation in the US, in contrast to the steadily declining contribution of coal-fired power plants, which dropped to 16% (*7*). In this case, CO_2_ capture from natural gas flue gas is in growing demand but remains challenging because of the low CO_2_ concentration, only about 4%, which makes the enrichment process complex and costly (*8*, *9*). Apart from the mature technologies for CO_2_ capture, such as distillation and absorption, advanced membrane technology stands out as particularly attractive because of its high energy efficiency and environmental friendliness (*10*–*12*).

Polymeric membranes are extensively investigated for CO_2_ separation, considering their low cost and high processability (*13*–*15*). The challenge of traditional polymeric membranes for practical use is the limited gas separation performance, ascribed to the intrinsic trade-off relationship between membrane permeability and selectivity, also known as Robeson upper bound (*16*, *17*). Recent advancements in enhancing CO_2_ transport of polymeric membranes include polymer modification with amine-functional groups or the incorporation of small-molecule amines into the polymer matrix (*18*, *19*). One specific example is polymeric facilitated transport membranes (FTMs), in which rapid CO_2_ transport is achieved through a reversible chemical reaction between CO_2_ and amine-functional groups within the membrane, rather than through the physical adsorption and diffusion processes typical of inert gases, such as N_2_ and CH_4_ (*20*, *21*). Despite their enhanced CO_2_ separation performance surpassing the Robeson upper bound (*22*, *23*), most state-of-the-art polymeric FTMs are limited by the intrinsic quick carrier saturation of CO_2_. That is, additional CO_2_ can only permeate through membrane via a poorly selective solution-diffusion route (*24*). This makes highly efficient one-step CO_2_ enrichment from lean CO_2_ sources, such as natural gas flue gas (4% of CO_2_), less feasible and practical (*25*).

Supported liquid-based membranes (SLMs), whose permselectivity is governed by fast gas transport carriers embedded within porous substrates (*26*), may have the potential to overcome the limitations of polymeric FTMs mentioned above (*27*–*29*). Despite advantages such as defect-free interface, self-recovery, and self-adaption (*30*), SLMs face serious stability issues due to liquid evaporation and pressure-driven leakage, which hinder their further industrial application in large scale (*31*). Ionic liquids (ILs), a type of molten salt at ambient temperature, have been introduced to address the thermal stability issues of SLMs, given their negligible vapor pressure (<10^−5^ Pa) even at elevated temperatures (*32*). Matsuyama and co-workers (*33*), for example, demonstrated that high CO_2_/N_2_ selectivity of supported IL membranes (SILMs) can be achieved even at 100°C by simply incorporating [P(C_4_)]_4_-type IL into a macroporous polytetrafluoroethylene support. In addition to thermal stability issues, another major challenge for SLMs is mechanical stability, as the CO_2_-philic liquid cannot be retained within the porous supports under prolonged pressure differences across them. Conventional methods for stabilizing SLMs include incorporating nanoparticles or negatively charged polymers into the existing matrix to enhance the liquid confinement, typically at the expense of CO_2_ carrier mobility, by narrowing gas transport channels or introducing electrostatic interactions (*34*, *35*). To further reduce CO_2_ transport resistance and improve stability of SILMs, efforts have also been made to confine ILs within submicrometer-thick, nanoconfined (~1-nm) laminar structures (*36*). Peng and co-workers (*37*–*39*) developed a series of 1-ethyl-3-methylimidazolium tetrafluoroborate ([Emim][BF_4_])–loaded laminated SILMs, achieving CO_2_/N_2_ selectivity ranging from 130 to 382. Although good CO_2_/N_2_ selectivity of these SILMs was retained, the mobility of ILs within them was greatly hindered by tortuous transport channels (*40*), exhibiting typically low CO_2_ permeance. The aforementioned observations offer vital insights into the design of applicable and processable SILMs with both high CO_2_ permeance and CO_2_/N_2_ selectivity, involving the use of highly CO_2_-selective IL carriers and ultrapermeable, nanoconfined structures that stabilize ILs.

**Fig. 1.**
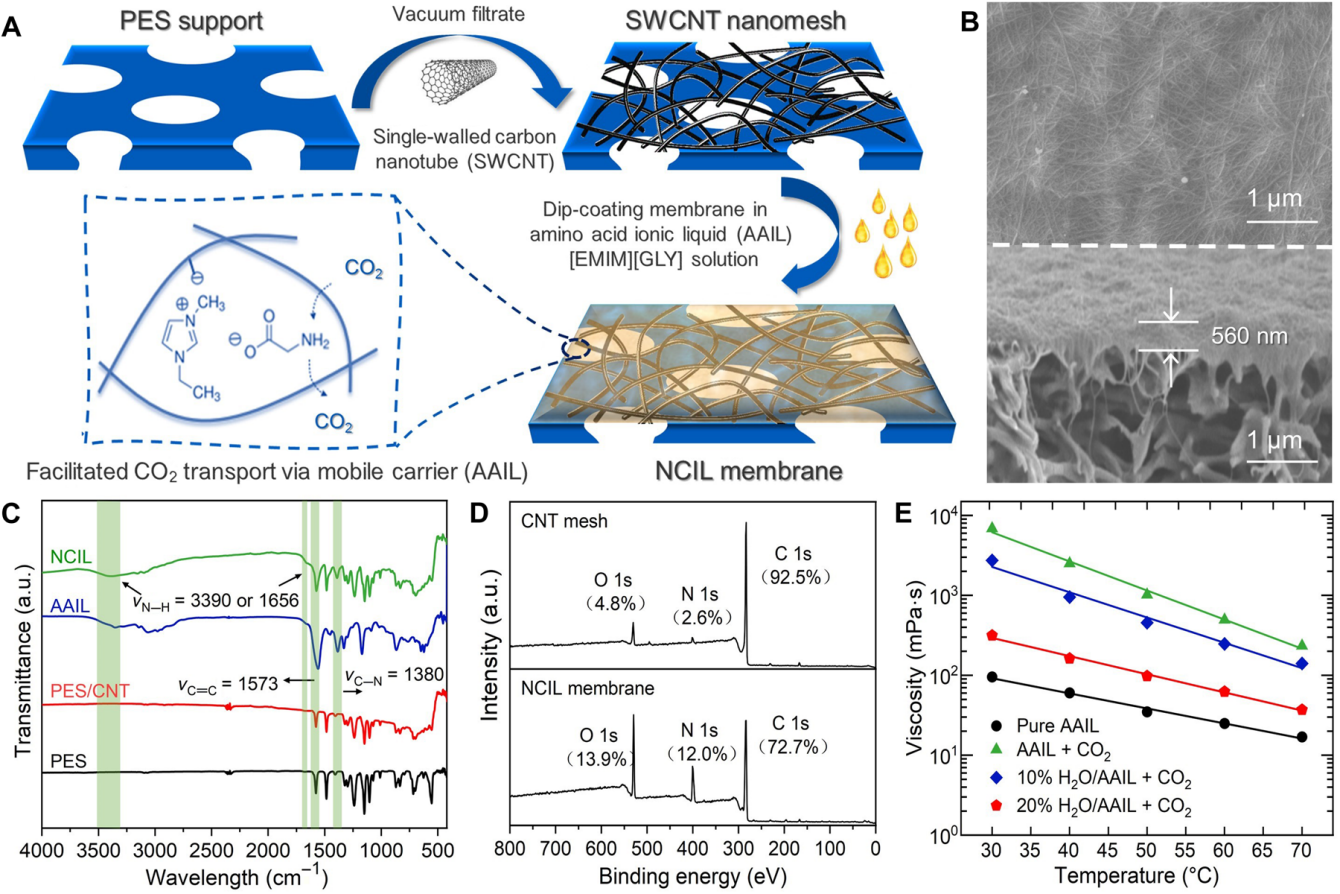
Conceptual design and characterization of NCIL membrane. (**A**) Schematics of the NCIL flat-sheet membrane fabrication procedure and facilitated CO_2_ transport via mobile carrier (AAIL). (**B**) Surface and cross-sectional scanning electron microscope (SEM) images of a representative NCIL membrane. (**C**) FTIR spectra of pristine PES support, CNT/PES membrane, AAIL ([Emim][Gly]), and NCIL membrane. a.u., arbitrary units. (**D**) XPS full spectra of CNT/PES membrane (top) and NCIL membrane (bottom). (**E**) Viscosity of pure [Emim][Gly] and [Emim][Gly]/H_2_O mixture before and after purging with CO_2_. NCIL membranes used for characterization were prepared with a CNT loading density of 60 mg m^−2^ and an IL solution of 150 mg ml^−1^ for dip coating.

In this work, we developed a scalable and facilely prepared nanoconfined IL membrane, referred to as the NCIL membrane, in which a highly CO_2_-selective amino acid IL (AAIL) was loaded into an ultrapermeable, nanoconfined single-walled carbon nanotube (SWCNT) network, as illustrated in Fig. 1, for highly efficient CO_2_ separation. Specifically, a nanoconfined structure composed of SWCNTs was first engineered to form an ultrathin, open, and uniform nanoconfined space (~10 nm), effectively restricting the IL while maintaining low transport resistance. The transport resistance and theoretical breakthrough pressure of nanoconfined SWCNT network was also investigated, demonstrating the necessity of ultrapermeable transport channel, combined with decent capillary force (~25 bar), for applicable and stable SILM construction. Subsequently, an AAIL, 1-ethyl-3-methylimidazolium glycine ([Emim][Gly]), was loaded into SWCNT mesh to form a continuous liquid layer and served as an effective CO_2_ mobile carrier owing to its intrinsic nature of fluidity and amine-rich functionality (*41*). We challenged the resulting NCIL membranes for CO_2_ capture from simulated natural gas flue gas, and the integration of ultrapermeable, nanoconfined SWCNT mesh with the CO_2_-selective AAIL enabled the stable, ultrahigh CO_2_/N_2_ selectivity up to 1132 and CO_2_ permeance as high as 1654 gas permeation units (GPU) for at least 100 hours at elevated temperatures and humid conditions, surpassing most state-of-the-art FTMs for CO_2_/N_2_ separation. To demonstrate scale-up potential, the optimized NCIL membrane was fabricated on a 75-cm^2^ hollow fiber substrate, showing excellent CO_2_/N_2_ separation performance to enrich CO_2_ from 4.2 to 98% in one step with a 54% stage cut. This work, therefore, demonstrated the processability and industrial prospect of SILMs and may provide guidance to design next-generation ultrapermeable and ultraselective SILMs for gas separation.

## RESULTS

### NCIL membrane fabrication and characterization

Figure 1A illustrates the facile preparation of a flat-sheet NCIL membrane via dip coating a CNT mesh into an IL solution (fig. S1). With the solvent evaporation, the capillary force provided by the CNT mesh is expected to draw the amine-functional IL into its nanopores for stable facilitated CO_2_ transport (*30*). Figure 1B shows a defect-free surface morphology and a membrane thickness of 560 nm for a representative NCIL membrane. Energy-dispersive spectroscopy (EDS) characterization further demonstrated the preferential concentration of IL, according to the N atoms derived from the IL, into the top CNT mesh to form a continuous layer rather than within the support (fig. S2).

The surface chemistry was further analyzed by Fourier transform infrared (FTIR) spectroscopy, as shown in Fig. 1C. Compared with the plain polyether sulfone (PES) support and the CNT mesh, new peaks appeared at 3390, 1656, and 1380 cm^−1^ for the NCIL membrane. This can be attributed to the primary amine and C─N bond from [Emim][Gly], which also has strong FTIR absorption peaks at these three wavelengths (*34*). Similarly, Fig. 1D shows that compared to the CNT mesh, x-ray photoelectron spectroscopy (XPS) spectrum of the NCIL membrane had more intensified nitrogen element peak due to the introduction of amine-functional groups. To better understand the immobilization and fluidity of IL in NCIL membrane, we measured the viscosity of [Emim][Gly] and [Emim][Gly]/H_2_O mixture. As shown in Fig. 1E, lower temperature induced viscosification of the AAIL, whereas the addition of a small amount of water considerably reduced its viscosity. Viscosity of the AAIL increased markedly by almost two orders of magnitude when CO_2_ was introduced, probably because of the strong intermolecular interaction between CO_2_ and the amine-functional groups (*42*). The viscosity and corresponding mobility of the AAIL in NCIL membrane are expected to depend strongly on the operation conditions, such as temperature, water content, and CO_2_, and their influence on the nanoconfined AAIL resistance to the pressure-driven flow in the CNT mesh will be further discussed in the gas separation section.

### Highly permeable CNT mesh construction for effective nanoconfinement

**Fig. 2.**
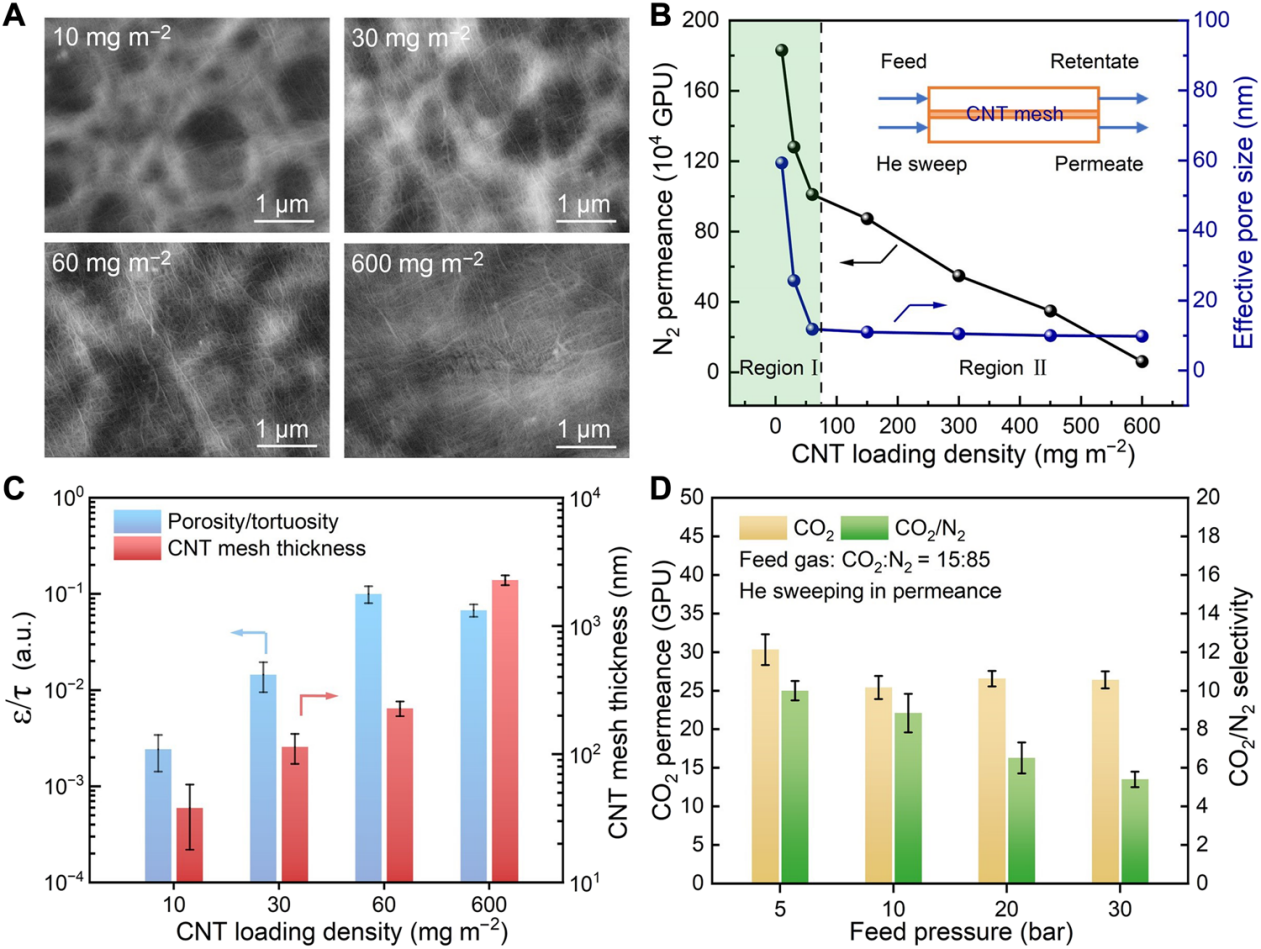
Effect of CNT loading density on physical and gas permeation properties of nanoconfined structure. (**A**) Surface SEM images of the CNT mesh deposited on the PES support with CNT loading density ranging from 10 to 600 mg m^−2^. (**B**) N_2_ permeation under 1.5-bar feed pressure at 70°C and effective pore size of the CNT mesh (inset: gas permeation measurement unit cell for N_2_; region I: pore size control region; region II: thickness control region; see detailed pore size calculation in the Supplementary Materials). (**C**) CNT mesh thickness and structure-relevant ε/τ factor as a function of CNT loading density. (**D**) Gas separation performance of NCIL membrane under high pressure, prepared with a CNT loading density of 60 mg m^−2^ and an IL solution of 150 mg ml^−1^ for dip coating (see detailed high-pressure characterization in the Supplementary Materials).

CNT nanomesh deposition and its nanostructures at different CNT loadings were first investigated before IL loading. Figure 2A shows the morphology of the CNT mesh corresponding to different CNT loading densities (CNT mass per membrane area). A defective mesh was obtained when the CNT loading density was below 10 mg m^−2^ (fig. S3), while further increasing the loading density by 3, 6, and 60 times resulted in denser and more compact nanostructures, making the substrate less visible. We also found that the thickness of the CNT mesh increased linearly with the CNT loading density (fig. S4), indicating the unchanged porosity of CNT mesh.

The microstructure of the resulting CNT mesh was further examined through molecular weight cutoff (MWCO) characterization, which is based on the rejection of polyethylene glycol (PEG) with varying molecular weights at 0.5-bar feed side pressure (see more details in the Supplementary Materials and fig. S5). As shown in Fig. 2B, the effective pore size of the CNT mesh markedly decreased from 59 to 11 nm with the increase in the CNT loading density from 10 to 60 mg m^−2^ and then only decreased slightly to 9.8 nm even after increasing CNT loading density by 10 times. The N_2_ permeation test was also conducted to reveal the ultralow gas transport resistance of the CNT network with N_2_ permeance up to 10^6^ GPU [1 GPU = 3.35 × 10^−10^ mol (m^2^·s·Pa)^−1^], and its corresponding change with different CNT loading densities (Fig. 2B). Two distinct regions of N_2_ permeance decline with increasing CNT loading density were identified, suggesting a transition in the nanostructure of the CNT mesh and a corresponding shift in transport resistance behavior. On the basis of the variations in pore size and thickness with increasing CNT loading density, the two distinct regions of N_2_ permeance decline can be attributed to a pore size–controlled region and a thickness-controlled region, respectively. Therefore, the optimal CNT mesh is expected at a CNT loading density of 60 mg m^−2^, the transition point of two regions, offering low transport resistance while effectively confining the IL for enhanced mechanical stability.

To further elucidate the inherent transport resistance of our nanoconfined networks, we introduced the structure-relevant ε/τ factor. As discussed in our previous work (*43*), the ε/τ factor was calculated on the basis of the pore-flow model, also known as Hagen-Poiseuille equation (*44*)

$$\text{Permeance}=\frac{J}{\Delta P}=\frac{\pi \varepsilon r_{p}^{2}}{8\mu \delta \tau }$$ (1)

where *J* is the flux, Δ*P* is the transmembrane pressure drop, ε is the surface porosity, *r*_p_ is the pore radius, μ is the solvent viscosity, δ is the membrane thickness, and τ is the transport channel tortuosity. In this case, high ε/τ factor implied high density of nanopores with low tortuosity and boosted transport property (*43*). Given the calculated pore radius from MWCO and the measured mesh thickness by scanning electron microscope (SEM), the ε/τ factor of CNT mesh was calculated using water permeation data at 0.5-bar pressure drop (fig. S6). Figure 2C shows a maximum ε/τ factor close to 0.1 at a CNT loading density of 60 mg m^−2^, outperforming other nanoconfined networks [see details in table S1, (*45*–*50*), and the Supplementary Materials].

Meanwhile, the nanoconfinement effectiveness of the CNT network was demonstrated via calculated theoretical breakthrough pressure, using the simplified Young-Laplace equation (*51*)

$$\text{Breakthrough Pressure}=\text{BP}=\frac{2\text{γcosθ}}{r_{p}}$$ (2)

where γ is the surface tension of liquid (*52*) and θ is the contact angle between the liquid and the pore surface (fig. S7). The calculated breakthrough pressures demonstrated the high-pressure tolerance of the CNT mesh–confined IL membrane of >25 bar (see the details in the Supplementary Materials), which was further validated through continuous pressurization and depressurization tests after loading IL into the optimized CNT mesh with a CNT loading density of 60 mg m^−2^.

Figure S8 (A to C) shows that the membrane thickness remained unchanged at 420 nm after pressurization under 10 bar of N_2_. An increase in N_2_ pressure up to 30 bar failed to displace the IL from the nanospace of the CNT mesh, indicating strong nanoconfinement. To demonstrate the continuity of the membrane-selective layer during pressurization, we also conducted single-gas permeation and mixed-gas separation tests of an NCIL membrane using the permeation test system shown in Fig. 2B. Figure S8D shows reversible N_2_ permeation during a pressurization and depressurization cycle. Figure 2D further demonstrates almost constant CO_2_ permeance even under 30 bar of pressurization with an increase in N_2_ permeance from 3 to 4.8 GPU, further implying the excellent mechanical stability of the confined IL-based membrane.

### IL regulation for defect-free NCIL membrane fabrication

**Fig. 3.**
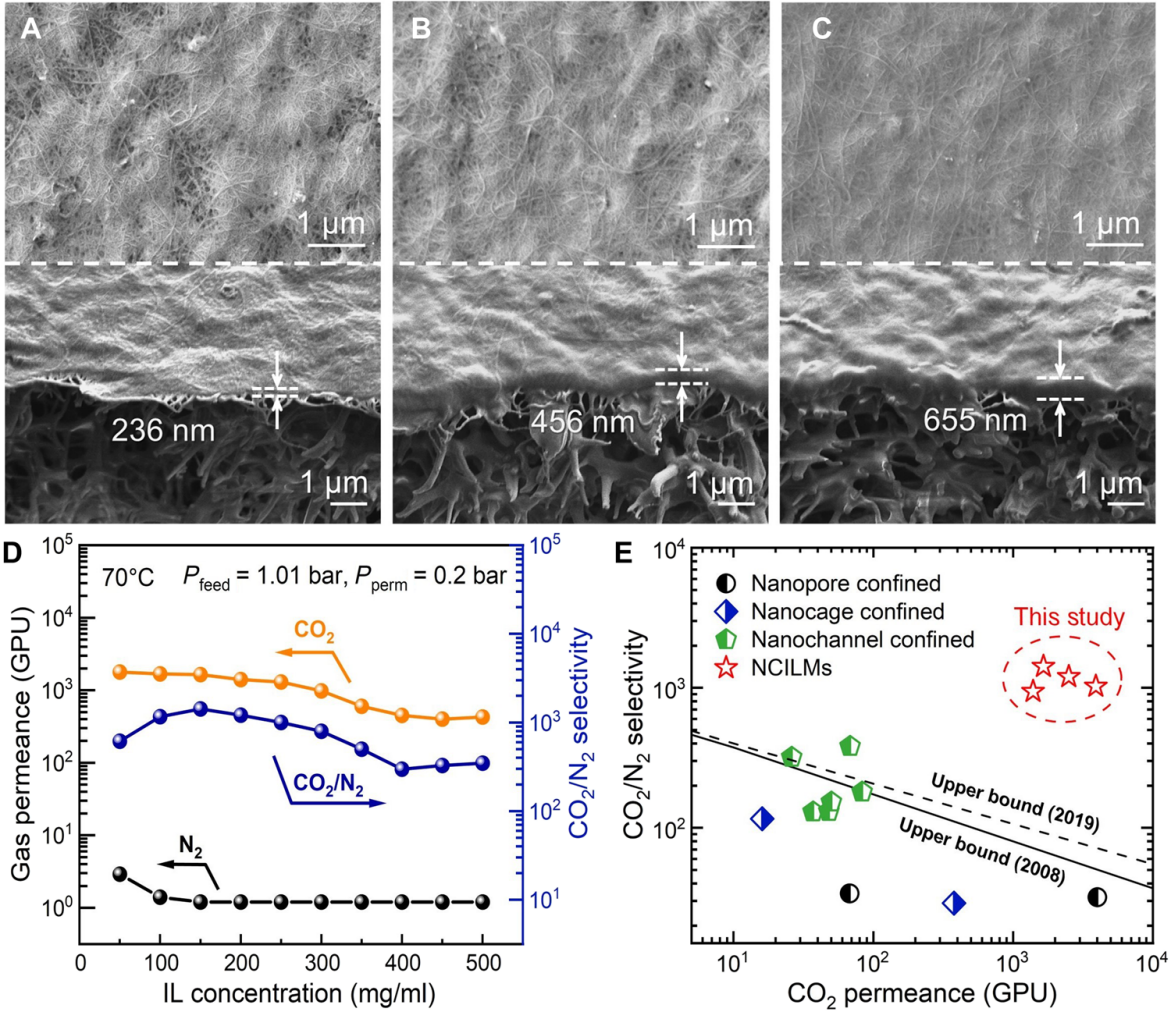
Effect of IL loading on morphology and gas separation performance of NCIL membrane. (**A** to **C**) Surface and cross-sectional SEM images of the NCIL membranes fabricated using varying IL concentrations of 50 mg ml^−1^ (A), 100 mg ml^−1^ (B) and 150 mg ml^−1^ (C), respectively. (**D**) Gas separation performance of the NCIL membranes as a function of IL concentration in the coating solution. (**E**) Gas separation performance comparison with reported NCIL membranes for CO_2_/N_2_ separation [data points are summarized in table S3, (*37*–*39*, *56*–*62*), and the Supplementary Materials]. The 2008 and 2019 upper bounds are shown as the black solid and dashed lines (*16*, *17*), respectively; membrane thickness was assumed to be 100 nm for converting permeability into permeance. Unless otherwise specified, NCIL membranes were prepared with a CNT loading density of 60 mg m^−2^ and an IL solution of 150 mg ml^−1^ for dip coating. NCIL membranes were tested using simulated flue gas (4.2% CO_2_, saturated H_2_O vapor, and balanced N_2_) under 1.01-bar feed pressure (absolute) and 0.2-bar (absolute) permeate side pressure at 70°C.

Building on the optimized, high-efficiency gas transport CNT mesh, AAIL was sequentially loaded into the nanoconfined space as a highly CO_2_-selective carrier, owing to its high CO_2_ solubility compared to N_2_ (*26*). Compared with nonselective, defective IL-loaded PES support (fig. S9 and table S2), Figure 3 (A to C) shows the gradual filling of IL into the CNT mesh led to a uniform and continuous layer, as IL concentration in the coating solution increased from 50 to 150 mg ml^−1^. The accumulated IL loading also swelled the CNT mesh, leading to an expanded NCIL membrane with a thickness from 236 to 656 nm. However, further introducing the IL loading by adding more IL in the coating solution did not linearly expand the CNT/IL selective layer; instead, it led to IL penetration into the support, blocking the transport pathways (figs. S10 and S11).

To further understand the influence of IL loading, we evaluated the gas separation performance of the NCIL membranes for simulated natural gas flue gas using a homemade system under vacuum operation mode (fig. S12). Specifically, NCIL membranes were tested using simulated flue gas composed of 4.2% CO_2_, saturated H_2_O vapor, and N_2_ as the balance gas, under a feed pressure of 1.01 bar (absolute pressure; consistent throughout the discussion), a permeate pressure of 0.2 bar, and at 70°C. As shown in Fig. 3D, CO_2_ permeance decreased gradually with the increase in IL loading, probably due to the increased CO_2_ transport resistance as suggested by Fig. 3 (A to C). On the other hand, N_2_ permeance initially decreased, apparently because of the pore filling of IL, and then remained nearly constant. Since the high loading of IL eventually saturated the polymeric support without further swelling the CNT mesh, the gas permeance of the NCIL membrane plateaued at high IL concentrations in the coating solution. Thus, the optimal IL concentration was identified as 150 mg ml^−1^ for forming a continuous and uniform selective layer with the highest CO_2_ permeance. A similar trade-off between gas permeance and selectivity was also found when tuning the CNT loading density. As shown in fig. S13, higher CNT density reduced CO_2_ permeance due to the limited carrier mobility but enhanced CO_2_/N_2_ selectivity by suppressing nonselective transport pathways, thus further confirming the optimal CNT loading density of 60 mg m^−2^, as identified in the previous section.

In Fig. 3E, we compared our optimized NCIL membranes with the reported NCIL membranes for CO_2_/N_2_ separation (table S3). Because of the highly efficient nanoconfinement of ILs, most IL-based membranes with ~1-nm pores/nanochannels exhibit high CO_2_/N_2_ selectivity but limited CO_2_ permeance. On the contrary, the NCIL membranes in this work demonstrated rapid CO_2_ transport property while maintaining high CO_2_/N_2_ selectivity, indicating the high gas separation efficiency of this NCIL-based design.

### Superior gas separation performance of the NCIL membrane

**Fig. 4.**
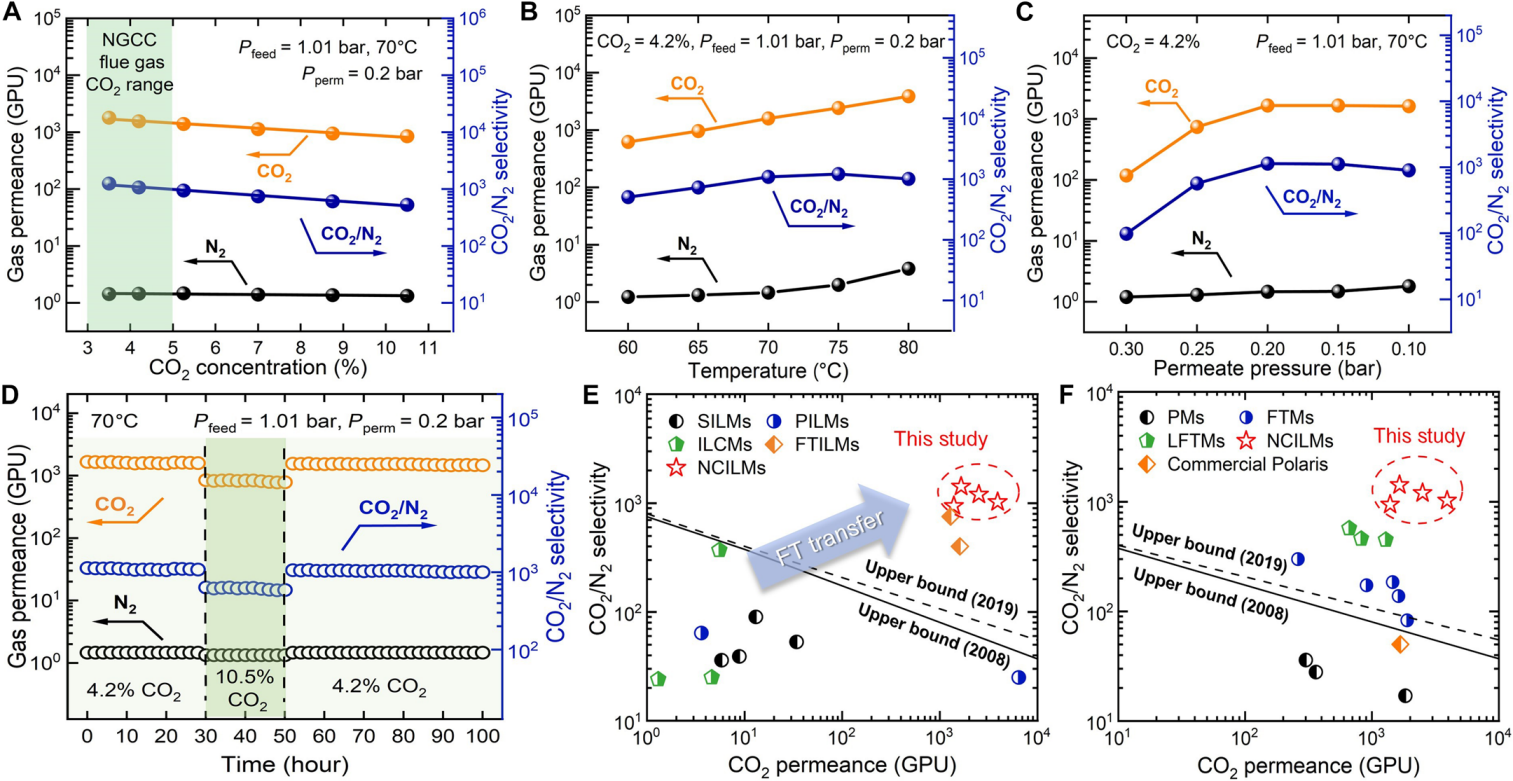
Superior and stable CO_2_/N_2_ mixed-gas separation performance of NCIL membrane. (**A**) Influence of CO_2_ concentration in the feed gas. (**B**) Influence of operation temperature. (**C**) Influence of permeate side pressure. (**D**) Long-term stability of the NCIL membrane. Green-highlighted zone indicates the change of CO_2_ concentration in the feed gas. (**E**) Gas separation performance comparison with the reported IL-based membranes for CO_2_/N_2_ separation [data points are summarized in table S4, (*63*–*73*), and the Supplementary Materials]. (**F**) Gas separation performance comparison with the reported thin film polymeric and facilitated transport membranes for CO_2_/N_2_ separation [data points are summarized in table S5, (*19*, *28*, *29*, *34*, *55*, *74*–*79*), and the Supplementary Materials]. The 2008 and 2019 upper bound limits are shown as black solid and dashed lines (*16*, *17*), respectively. Membrane thickness was assumed to be 100 nm for converting permeability into permeance. The NCIL membrane was prepared with a CNT loading density of 60 mg m^−2^ and an IL solution of 150 mg ml^−1^ for dip coating. Unless otherwise specified, the NCIL membrane was tested using simulated flue gas composed of 4.2% CO_2_, saturated H_2_O vapor, and balanced N_2_ under 1.01-bar feed pressure (absolute) and 0.2-bar (absolute) permeate side pressure at 70°C. FT, facilitated transport.

To further explore the separation capability of the optimized NCIL membrane, we investigated the influence of the operation conditions (Fig. 4). As shown in Fig. 4A, CO_2_ permeance declined almost exponentially from 1784 to 845 GPU when feed CO_2_ concentration increased from 3.5 to 10.5%. The characteristic CO_2_ saturation behavior of gas transport agents under elevated CO_2_ partial pressure reflects the facilitated transport mechanism enabling CO_2_ permeation through the NCIL membrane (*21*, *22*). In contrast, N_2_ permeance only decreased slightly with the decrease in N_2_ partial pressure resulting from the increased CO_2_ concentration. This can be attributed to the solution-diffusion mechanism that inert gases usually follow (*20*). Thus, the CO_2_/N_2_ selectivity also decreased exponentially with the increase in CO_2_ concentration, following the same trend as the change in CO_2_ permeance. The characteristic permeance trend of the facilitated transport gas (CO_2_) under elevated feed pressure is more pronounced in fig. S14, where CO_2_ permeance decreased from 1548 to 519 GPU as the feed pressure increased from 1.01 bar to 2 bar, with constant CO_2_ and N_2_ concentrations in the feed. In summary, NCIL membranes exhibited superior separation performance, with a CO_2_ permeance of 1548 GPU and a CO_2_/N_2_ selectivity of 1066 within 3 to 5% CO_2_ concentration and at 1.01-bar feed pressure, highlighting their potential as ideal candidates for CO_2_ capture from natural gas flue gas (*9*).

The dependence of temperature and permeate side pressure on CO_2_ separation performance of the NCIL membrane was then examined. As shown in Fig. 4B, increasing the temperature from 60° to 80°C, a typical temperature range for CO_2_ capture from natural gas flue gas (*8*), led to an exponential increase in CO_2_ permeance from 619 to 3857 GPU; the calculated activation energy under fully saturated conditions was 86.2 kJ mol^−1^ (fig. S15). On the other hand, N_2_ permeance increased from 1.23 to 3.83 GPU after 75°C, resulting in a slight drop of CO_2_/N_2_ selectivity. This change can be attributed to the improved mobility of the IL, specifically, the decreased viscosity under elevated temperature (Fig. 1E), which facilitated faster CO_2_ transport and slightly “loosened” the NCIL membrane structure. Figure 4C shows the membrane separation performance at different permeate side pressures. Lower permeate side pressure increased the transmembrane driving force for gases and promoted CO_2_ transport, with CO_2_ permeance increasing from 118 to 1654 GPU. Unlike CO_2_ having high binding energy with amine-based carriers, N_2_ exhibited negligible intermolecular interaction with AAIL (*53*). As a result, only negligible change in N_2_ permeance was observed with decreasing permeate pressure, while the CO_2_/N_2_ selectivity followed a trend similar to that of CO_2_ permeance, reaching a maximum selectivity of 1132 at a permeate side pressure of 0.2 bar.

The long-term stability of the NCIL membrane for CO_2_/N_2_ gas mixture separation was evaluated under simulated flue gas conditions. As indicated in Fig. 4D, the NCIL membrane was stable for the first 30 hours and showed a CO_2_ permeance of 1654 GPU and a CO_2_/N_2_ selectivity of 1132 for CO_2_ capture from simulated natural gas flue gas containing 4.2% CO_2_. Then, the CO_2_ concentration was increased to 10.5% to mimic CO_2_ capture from coal-fired flue gas (*28*), and the NCIL membrane exhibited a stable CO_2_ permeance of 820 GPU and a CO_2_/N_2_ selectivity of 616 during 20-hour continuous testing. The separation performance of the NCIL membrane recovered its initial performance after switching CO_2_ concentration to 4.2% and remained stable for 50 hours. Figure 4E compares the NCIL membranes with traditional IL-based membranes reported in the literature for CO_2_/N_2_ separation (table S4). Because of the combination of rapid gas transport channels and facilitated transport characteristics, the NCIL membranes surpass the Robeson upper bound and outperform traditional IL-based membranes. Furthermore, the NCIL membranes demonstrated substantially superior CO_2_/N_2_ separation performance compared to most state-of-the-art polymeric and facilitated transport–based membranes (Fig. 4F and table S5), particularly outperforming the commercial Polaris membrane, showcasing that this class of membranes may serve as the next generation of rapid CO_2_ transport membranes for capturing CO_2_ from lean-concentration point sources.

### Natural gas flue gas separation using scaled-up NCIL membrane

**Fig. 5.**
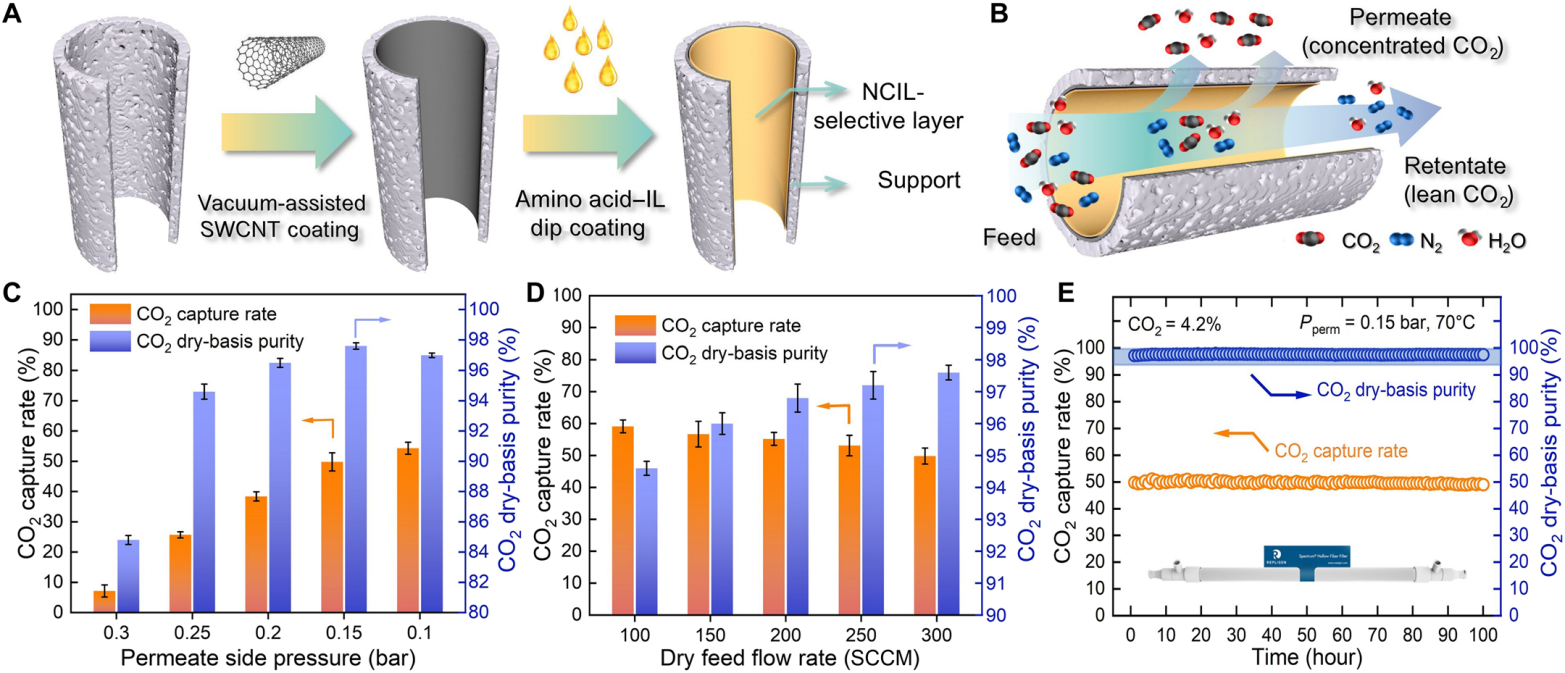
Demonstration of NCIL membrane scaling up and industrial natural gas flue gas separation. (**A**) Schematics of the hollow fiber NCIL membrane fabrication procedure. (**B**) Illustration of CO_2_ transport through the resulting membrane. The CO_2_/N_2_ mixed-gas separation performance of the scaled-up NCIL membrane as a function of membrane operation conditions. (**C**) Influence of permeate side pressure. (**D**) Influence of dry feed flow rate. (**E**) Long-term stability of NCIL membrane (inset: photo of a 75-cm^2^ hollow fiber module). The NCIL membrane was prepared with a CNT loading density of 60 mg m^−2^ and an IL solution of 150 mg ml^−1^ for dip coating. Unless otherwise specified, the membrane was tested using simulated flue gas composed of 4.2% CO_2_ (6% dry basis), saturated H_2_O vapor, and balanced N_2_ under 1.01-bar feed pressure (absolute) and 0.15-bar (absolute) permeate side pressure at 70°C, with feed gas flow rate of 200 standard cubic centimeter per minute (SCCM).

To explore the potential for large-scale application, we further developed the NCIL membrane by coating it onto the inner surface of a 75-cm^2^ hollow fiber PES support, leveraging the high packing density and scalability of hollow fiber modules (*54*). A similar membrane fabrication procedure as that of the flat-sheet membrane was followed, including vacuum-assistant CNT coating and IL solution dip coating (Fig. 5A; details of hollow fiber NCIL membrane preparation and morphology in figs. S16 and S17). The resulting defect-free NCIL hollow fiber membrane was tested in a modified laboratory-made system (fig. S18), using 1.01-bar simulated natural gas flue gas (4.2% CO_2_, saturated H_2_O vapor, and balanced N_2_) as the feed gas under 0.15-bar permeate side pressure outside the hollow fiber membrane (Fig. 5B) at 70°C.

Apart from ultrahigh CO_2_ permeance and CO_2_/N_2_ selectivity (table S6; see calculation details in the Supplementary Materials), CO_2_ capture rate and CO_2_ dry-basis purity in permeate side were also evaluated for industrial production purposes. As shown in Fig. 5C, the NCIL membrane demonstrated its capability to enrich CO_2_ from 4.2 to 98% in a single step, owing to its ultrahigh CO_2_/N_2_ selectivity. Decreasing the permeate side pressure from 0.3 to 0.15 bar enabled larger driving force for rapid CO_2_ transport, thus improving the CO_2_ capture rate from 7.1 to 54.1% and the CO_2_ dry-basis purity from 84.1 to 97.6%. Meanwhile, the separation performance of the NCIL membrane could be further enhanced by mitigating concentration polarization, defined as the accumulation of slower-permeating species (N_2_) near the membrane surface due to the depletion of the preferentially permeating species (CO_2_), which is particularly pronounced in fast CO_2_ transport, highly selective membranes operating at a stage cut (*55*). The resulting decrease in CO_2_ and increase in N_2_ driving forces typically lead to reduced CO_2_/N_2_ selectivity and diminished CO_2_ flux. In this case, increasing feed flow rate facilitates CO_2_ bulk diffusion toward the membrane surface and thus increases CO_2_ flux. As shown in Fig. 5D, increasing the feed gas flow rate from 150 to 300 standard cubic centimeters per minute (SCCM) improved CO_2_ dry-basis purity from 94.6 to 97.6%, while the CO_2_ capture rate decreased from 59.1 to 49.8%, indicating the enhanced CO_2_ separation capability at the expense of capture efficiency.

The long-term stability test of the scaled-up hollow fiber membrane was performed, as shown in Fig. 5E. The results revealed only a slight decrease in CO_2_ capture rate (from 50.3 to 49.0%) and a relatively constant CO_2_ dry-basis purity (97.6 ± 0.2%) over more than 100 hours under simulated natural gas flue gas separation conditions. Apart from that, CO_2_ separation performance was also evaluated using simulated coal-fired flue gas (fig. S19), showing stable separation performance for 100 hours with CO_2_ dry-basis purity of 97.9 ± 0.1 and 97.5 ± 0.5% at 65° and 70°C, respectively. Given the practical and scalable fabrication of the NCIL membrane, this work presents a promising candidate for highly efficient CO_2_ capture from various point sources.

## DISCUSSION

In summary, we developed a scalable, facilely prepared, and defect-free NCIL membrane by simply incorporating CO_2_-selective AAIL into an ultrapermeable, nanoconfined SWCNT network without further modification. The ultrapermeable SWCNT nanoconfined structure was optimized to provide minimum gas transport resistance and maximum confinement for further IL stabilization. The investigation of transport resistance and breakthrough pressure further demonstrated superiority of the SWCNT network, which combines high ε/τ factor with strong confinement force. Specifically, continuous pressurization test verified the effectiveness of nanoconfinement of the SWCNT mesh even under 30 bar of pressure drop. On the other hand, CO_2_-philic AAIL was incorporated within the SWCNT nanomesh, not only as mobile carriers for fast CO_2_ facilitated transport but also as a self-stabilizer, as validated by the viscosification process between AAIL and CO_2_. By marrying the merits of SWCNT mesh and AAIL, the NCIL membrane showed ultrahigh CO_2_/N_2_ selectivity up to 1132 with CO_2_ permeance of 1654 GPU for CO_2_ capture from simulated natural gas flue gas, surpassing most state-of-the-art FTMs. Membrane stability was confirmed for at least 100 hours under elevated temperature and humid conditions. The potential of the NCIL membrane for large-scale application was further demonstrated by the first reported highly efficient one-step CO_2_ enrichment from 4.2 to 98%, achieving a stage cut of 54%. Given the facile and scalable preparation of our NCIL membranes, this work offers an innovative perspective for designing highly effective nanoconfined transport structures for SILMs in gas separation and suggests a promising alternative route for transforming solvent absorption technology into membrane-based technology for CO_2_ capture.

## MATERIALS AND METHODS

### Materials

SWCNT powder (optical density < 3 nm, length = 5 μm, purity > 85%; TCI), sodium dodecyl benzenesulfonate (SDBS; 99%; TCI), [Emim][Gly] (>98%; BLDpharm) were used as received. PES (30/100/450 nm, 51 mm in diameter) was purchased from Steritech Corporation. PES hollow fiber substrate (75 cm^2^, 300-kDa MWCO, 1-mm inner diameter) was purchased from Repligen Corporation. PEG (molecular weight, 600 Da and 2, 10, and 35 kDa) and polyethylene oxide (molecular weight, 100 and 300 kDa) were purchased from Sigma-Aldrich. Premixed gas (15% CO_2_/85% N_2_) and pure gas (99.9 mol % N_2_, 99.9 mol % CO_2_, and 99.99 mol % He) for membrane permeability and selectivity evaluation were purchased from Airgas.

### CNT dispersion and coating solution preparation

CNT powder (0.1 mg) was added into 1 liter of as-prepared SDBS solution [1 mg of SDBS per milliliter of deionized (DI) water] and dispersed via ultrasonication (Thermo Fisher Scientific, S450) for 1 hour. Then, CNT dispersion after sonication was centrifuged at 10,000 rounds per minute (rpm) for 25 min, and the supernatant was collected as final CNT dispersion with CNT concentration of 0.06 mg ml^−1^. Last, CNT dispersion was further diluted into DI water to make a CNT-coating solution of 1 μg ml^−1^.

### Flat sheet NCIL membrane fabrication

A controlled volume of CNT-coating solution was vacuum filtrated onto a flat-sheet PES substrate (450-nm pore size) to fabricate the CNT nanomesh. The resulting CNT nanomesh was dried in oven at 70°C for 1 hour. NCIL flat-sheet membrane was prepared using a dip-coating method. The coating solution was prepared by adding controlled amount of IL ([Emim][Gly], 98%) into DI water (10 ml). Then, the dip-coating solution was stirred for 10 min. The dip-coating procedure was illustrated in fig. S1. As-prepared CNT nanomesh on PES substrate was cut into 2 cm by 4 cm flat sheet and taped onto a microscopic slide and then fixed onto the rod of dip coater. The moving speed of the dip coater rod was set to be 10^−3^ m s^−1^. During the dip-coating process, the CNT nanomesh was completely immersed in IL-coating solution for 1 s and then taken out of solution following the pre-set program. Last, the NCIL membrane was transferred to a petri dish and dried in oven at 70°C for 1 hour.

### Scale-up fabrication of hollow fiber NCIL membranes

CNT nanomesh on hollow fiber PES substrate was first prepared using a modified vacuum-assisted coating system as illustrated in fig. S16A. We first introduced DI water to fill up PES hollow fiber substrate to remove glycols within the substrate, and the pumping rate of DI water was controlled by a syringe pump. Then, the CNT solution was infused into the washed support until all the air bubbles inside the fibers were removed. Vacuum pressure (0.2 bar) was sequentially applied in the permeate side of hollow fiber module, and CNT-coating solution started to be pulled into hollow fiber. After complete consumption of the CNT-coating solution, vacuum was maintained on the permeate side for an additional 30 min to remove DI water from inside the module. Last, the resulting CNT nanomesh on PES hollow fiber substrate was taken out of the coating system and dried in the oven at 70°C overnight.

NCIL hollow fiber membrane was also prepared via the dip-coating method as shown in fig. S16B. The dip-coating solution was prepared by adding 12 g of [Emim][Gly] into 60 ml of DI water with stirring for 10 min. At the start of the dip-coating process, the AAIL solution was pumped into CNT nanomesh–coated hollow fibers with a controlled pumping rate of 3 ml min^−1^. After complete filling-up of the hollow fibers with IL solution, the syringe was removed to allow IL solution to slowly flow out of the hollow fibers by gravity. Last, NCIL hollow fiber membrane was taken out of the coating system and dried in the oven at 70°C overnight.

### Characterization

The surface and cross-sectional morphology of membranes were characterized by focused ion beam SEM Carl Zeiss AURIGA. Element distributions of NCIL membranes were analyzed via EDS (Hitachi, SU70). FTIR spectroscopy (Bruker VERTEX 70) has been performed to investigate the IL incorporation within NCIL membranes. NCIL membrane structure and chemical properties were further investigated using XPS (AXIS Ultra DLD, Kratos Analytical). MWCO of CNT mesh was characterized by gel permeation chromatography (Agilent 1260 Infinity II, Column: Agilent OligoPore, PL1113-6520) via applying PEG filtration through CNT/PES membranes (See details in the Supplementary Materials).

### Single- and mixed-gas permeation measurement

Single-gas permeation test was conducted using sweep system as shown in fig. S12. Flat-sheet membrane was cut into 2 cm by 4 cm small pieces and put inside the stainless-steel membrane module with an effective membrane area of 0.23 cm^2^. Feed gas pressure was adjusted directly by gas regulator of CO_2_ or N_2_ cylinder. Helium was used as sweep and carrier gas in permeate side with a flow rate of 60 ml min^−1^ controlled by mass flow controller (Brooks, 5850S). Permeate side gas was carried by helium and sent to gas chromatography (Agilent, GC8890) for gas composition analysis. The permeation temperature was controlled by air-forced oven. Gas mixture separation test was conducted using the same laboratory-built vacuum system as shown in fig. S12. For flat-sheet NCIL membrane, the same membrane stainless steel module in the single gas permeation test was used. The dry CO_2_/N_2_ gas mixture was first generated and controlled by mass flow controllers (Brooks, 5850S). Water vapor was then introduced via humidifier into dry CO_2_/N_2_ gas mixture to make up feed gas with different water vapor compositions. Mass flow controller in the retentate side of membrane was used to adjust membrane feed side pressure via controlling the retentate side gas flow rate. Permeate side pressure was controlled by needle valve between membrane permeate outlet and vacuum pump. Last, permeate gas was carried by helium (60 ml min^−1^) and sent to gas chromatography (Agilent, GC8890) for gas composition analysis. The permeation temperature was controlled by air-forced oven and details of the gas permeation calculations are provided in the Supplementary Materials.

For gas mixture permeation measurement of scaled-up (75 cm^2^) hollow fiber NCIL membrane, the system setup was similar to that for flat-sheet membrane, as shown in fig. S18. NCIL hollow fiber membrane was placed vertically within oven, and simulated natural gas flue gas was introduced as feed gas into bottom side of hollow fiber. Water condenser inside chiller was assembled before vacuum pump to collect water vapor in permeate gas. Then the water vapor–free permeate gas was carried by helium and sent to gas chromatography (Agilent, GC8890) for gas composition analysis. The details of the gas permeation calculations are provided in the Supplementary Materials.

## Supplementary Materials

### This PDF file includes:

Supplementary Text Figs. S1 to S19 Tables S1 to S6 References

## References

[1] M. R. Raupach, C. Le Quéré, G. P. Peters, J. G. Canadell, Anthropogenic CO_2_ emissions. Nat. Clim. Change 3, 603–604 (2013).

[2] S. I. Seneviratne, M. G. Donat, A. J. Pitman, R. Knutti, R. L. Wilby, Allowable CO_2_ emissions based on regional and impact-related climate targets. Nature 529, 477–483 (2016). 26789252 10.1038/nature16542

[3] M. E. Boot-Handford, J. C. Abanades, E. J. Anthony, M. J. Blunt, S. Brandani, N. Mac Dowell, J. R. Fernández, M. C. Ferrari, R. Gross, J. P. Hallett, R. S. Haszeldine, P. Heptonstall, A. Lyngfelt, Z. Makuch, E. Mangano, R. T. J. Porter, M. Pourkashanian, G. T. Rochelle, N. Shah, J. G. Yao, P. S. Fennell, Carbon capture and storage update. Energy Environ. Sci. 7, 130–189 (2014).

[4] S. Chu, Carbon capture and sequestration. Science 325, 1599 (2009). 19779157 10.1126/science.1181637

[5] Y. Li, X. Yang, E. Du, Y. Liu, S. Zhang, C. Yang, N. Zhang, C. Liu, A review on carbon emission accounting approaches for the electricity power industry. Appl. Energy 359, 122681 (2024).

[6] Q. Zhou, A. Manuilova, J. Koiwanit, L. Piewkhaow, M. Wilson, C. W. Chan, P. Tontiwachwuthikul, A comparative of life cycle assessment of post-combustion, pre-combustion and oxy-fuel CO_2_ capture. Energy Procedia 63, 7452–7458 (2014).

[7] US Energy Information Administration, “Electric power monthly” (2025); https://www.eia.gov/electricity/monthly/.

[8] R. L. Siegelman, P. J. Milner, E. J. Kim, S. C. Weston, J. R. Long, Challenges and opportunities for adsorption-based CO_2_ capture from natural gas combined cycle emissions. Energy Environ. Sci. 12, 2161–2173 (2019). 33312228 10.1039/c9ee00505f PMC7731587

[9] M.-B. Hägg, A. Lindbråthen, CO_2_ capture from natural gas fired power plants by using membrane technology. Ind. Eng. Chem. Res. 44, 7668–7675 (2005).

[10] R. W. Baker, B. T. Low, Gas separation membrane materials: A perspective. Macromolecules 47, 6999–7013 (2014).

[11] D. L. Gin, R. D. Noble, Designing the next generation of chemical separation membranes. Science 332, 674–676 (2011). 21551053 10.1126/science.1203771

[12] R. Hou, C. Fong, B. D. Freeman, M. R. Hill, Z. Xie, Current status and advances in membrane technology for carbon capture. Sep. Purif. Technol. 300, 121863 (2022).

[13] Y. Ji, M. Zhang, K. Guan, J. Zhao, G. Liu, W. Jin, High-performance CO_2_ capture through polymer-based ultrathin membranes. Adv. Funct. Mater. 29, 1–9 (2019).

[14] S. Wang, X. Li, H. Wu, Z. Tian, Q. Xin, G. He, D. Peng, S. Chen, Y. Yin, Z. Jiang, M. D. Guiver, Advances in high permeability polymer-based membrane materials for CO_2_ separations. Energy Environ. Sci. 9, 1863–1890 (2016).

[15] Z. Dai, L. Ansaloni, L. Deng, Recent advances in multi-layer composite polymeric membranes for CO_2_ separation: A review. Green Energy Environ. 1, 102–128 (2016).

[16] B. Comesaña-Gándara, J. Chen, C. G. Bezzu, M. Carta, I. Rose, M.-C. Ferrari, E. Esposito, A. Fuoco, J. C. Jansen, N. B. McKeown, Redefining the Robeson upper bounds for CO_2_/CH_4_ and CO_2_/N_2_ separations using a series of ultrapermeable benzotriptycene-based polymers of intrinsic microporosity. Energy Environ. Sci. 12, 2733–2740 (2019).

[17] L. M. Robeson, The upper bound revisited. J. Membr. Sci. 320, 390–400 (2008).

[18] M. Sandru, M. Prache, T. Macron, L. Căta, M. G. Ahunbay, M.-B. Hägg, G. Maurin, M. Barboiu, Rubbery organic frameworks (ROFs) toward ultrapermeable CO_2_-selective membranes. Sci. Adv. 10, eadq5024 (2025). 10.1126/sciadv.adq5024 PMC11559614 39536097

[19] Y. Chen, L. Zhao, B. Wang, P. Dutta, W. S. Winston Ho, Amine-containing polymer/zeolite Y composite membranes for CO_2_/N_2_ separation. J. Membr. Sci. 497, 21–28 (2016).

[20] J. G. Wijmans, R. W. Baker, The solution-diffusion model: A review. J. Membr. Sci. 107, 1–21 (1995).

[21] Z. Tong, W. S. W. Ho, Facilitated transport membranes for CO_2_ separation and capture. Sep. Sci. Technol. 52, 156–167 (2017).

[22] T.-Y. Chen, X. Deng, L.-C. Lin, W. S. W. Ho, New sterically hindered polyvinylamine-containing membranes for CO_2_ capture from flue gas. J. Membr. Sci. 645, 120195 (2022).

[23] M. Sandru, E. M. Sandru, W. F. Ingram, J. Deng, P. M. Stenstad, L. Deng, R. J. Spontak, An integrated materials approach to ultrapermeable and ultraselective CO_2_ polymer membranes. Science 376, 90–94 (2022). 35357934 10.1126/science.abj9351

[24] Y. Han, W. S. W. Ho, Mitigated carrier saturation of facilitated transport membranes for decarbonizing dilute CO_2_ sources: An experimental and techno-economic study. J. Membr. Sci. Lett. 2, 100014 (2022).

[25] B. Belaissaoui, D. Willson, E. Favre, Membrane gas separations and post-combustion carbon dioxide capture: Parametric sensitivity and process integration strategies. Chem. Eng. J. 211-212, 122–132 (2012).

[26] A. Chamoun-Farah, A. N. Keller, M. Y. Balogun, L. M. Cañada, J. F. Brennecke, B. D. Freeman, Amine functionalized supported ionic liquid membranes (SILMs) for CO_2_/N_2_ separation. J. Membr. Sci. 702, 122758 (2024).

[27] L. Wang, Y. Zhou, S. Zha, S. Zhang, J. Jin, An ionic covalent organic framework membrane with confined mobile carriers for stable and efficient carbon dioxide capture. ACS Sustain. Chem. Eng. 12, 18475–18484 (2024).

[28] H. Li, S. Zhang, B. Sengupta, H. Li, F. Wang, S. Li, M. Yu, Polystyrene sulfonate (PSS) stabilized polyethylenimine (PEI) membranes fabricated by spray coating for highly effective CO_2_/N_2_ separation. J. Membr. Sci. 657, 120617 (2022).

[29] F. Zhou, H. N. Tien, Q. Dong, W. L. Xu, H. Li, S. Li, M. Yu, Ultrathin, ethylenediamine-functionalized graphene oxide membranes on hollow fibers for CO_2_ capture. J. Membr. Sci. 573, 184–191 (2019).

[30] Z. Sheng, J. Zhang, J. Liu, Y. Zhang, X. Chen, X. Hou, Liquid-based porous membranes. Chem. Soc. Rev. 49, 7907–7928 (2020). 32705106 10.1039/d0cs00347f

[31] P. Bernardo, E. Drioli, G. Golemme, Membrane gas separation: A review/state of the art. Ind. Eng. Chem. Res. 48, 4638–4663 (2009).

[32] N. V. Plechkova, K. R. Seddon, Applications of ionic liquids in the chemical industry. Chem. Soc. Rev. 37, 123–150 (2008). 18197338 10.1039/b006677j

[33] S. Kasahara, E. Kamio, T. Ishigami, H. Matsuyama, Effect of water in ionic liquids on CO_2_ permeability in amino acid ionic liquid-based facilitated transport membranes. J. Membr. Sci. 415-416, 168–175 (2012). 10.1039/c2cc17380h 22374137

[34] S. Zhang, H. Li, H. Li, B. Sengupta, S. Zha, S. Li, M. Yu, Negative charge confined amine carriers within the nanowire network for stable and efficient membrane carbon capture. Adv. Funct. Mater. 30, 1–7 (2020).

[35] F. Zhou, H. N. Tien, Q. Dong, W. L. Xu, B. Sengupta, S. Zha, J. Jiang, D. Behera, S. Li, M. Yu, Novel carbon-based separation membranes composed of integrated zero- and one-dimensional nanomaterials. J. Mater. Chem. A 8, 1084–1090 (2020).

[36] W. Luo, C. Wang, M. Jin, F. Li, H. Li, Z. Zhang, X. Zhang, Y. Liang, G. Huang, T. Zhou, Research progress on nanoconfined ILs in two-dimensional composite membranes for CO_2_ capture. Sep. Purif. Technol. 330, 125406 (2024).

[37] W. Ying, J. Cai, K. Zhou, D. Chen, Y. Ying, Y. Guo, X. Kong, Z. Xu, X. Peng, Ionic liquid selectively facilitates CO_2_ transport through graphene oxide membrane. ACS Nano 12, 5385–5393 (2018). 29874039 10.1021/acsnano.8b00367

[38] D. Chen, W. Wang, W. Ying, Y. Guo, D. Meng, Y. Yan, R. Yan, X. Peng, CO_2_-philic WS_2_ laminated membranes with a nanoconfined ionic liquid. J. Mater. Chem. A 6, 16566–16573 (2018).

[39] D. Chen, W. Ying, Y. Guo, Y. Ying, X. Peng, Enhanced gas separation through nanoconfined ionic liquid in laminated MoS_2_ membrane. ACS Appl. Mater. Interfaces 9, 44251–44257 (2017). 29191003 10.1021/acsami.7b15762

[40] C. Shao, W. L. Ong, J. Shiomi, A. J. H. McGaughey, Nanoconfinement between graphene walls suppresses the near-wall diffusion of the ionic liquid [BMIM][PF_6_]. J. Phys. Chem. B 125, 4527–4535 (2021). 33885322 10.1021/acs.jpcb.1c02562

[41] F. Wang, D. K. Behera, K. Friedman, J. Lyu, S. Li, M. Yu, Heterogeneous facilitated transport membrane via ionic liquid-mediated interfacial polymerization for CO_2_ separation. Adv. Funct. Mater. 35, 2422445 (2025).

[42] K. E. Gutowski, E. J. Maginn, Amine-functionalized task-specific ionic liquids: A mechanistic explanation for the dramatic increase in viscosity upon complexation with CO_2_ from molecular simulation. J. Am. Chem. Soc. 130, 14690–14704 (2008). 18847198 10.1021/ja804654b

[43] B. Sengupta, Q. Dong, R. Khadka, D. K. Behera, R. Yang, J. Liu, J. Jiang, P. Keblinski, G. Belfort, M. Yu, Carbon-doped metal oxide interfacial nanofilms for ultrafast and precise separation of molecules. Science 381, 1098–1104 (2023). 37676942 10.1126/science.adh2404

[44] W. R. Bowen, J. S. Welfoot, Modelling of membrane nanofiltration—Pore size distribution effects. Chem. Eng. Sci. 57, 1393–1407 (2002).

[45] S. Karan, Z. Jiang, A. G. Livingston, Sub–10 nm polyamide nanofilms with ultrafast solvent transport for molecular separation. Science 348, 1347–1351 (2015). 26089512 10.1126/science.aaa5058

[46] Y. Jin, Q. Song, N. Xie, W. Zheng, J. Wang, J. Zhu, Y. Zhang, Amidoxime-functionalized polymer of intrinsic microporosity (AOPIM-1)-based thin film composite membranes with ultrahigh permeance for organic solvent nanofiltration. J. Membr. Sci. 632, 119375 (2021).

[47] Q. Lan, C. Feng, K. Ou, Z. Wang, Y. Wang, T. Liu, Phenolic membranes with tunable sub-10-nm pores for nanofiltration and tight-ultrafiltration. J. Membr. Sci. 640, 119858 (2021).

[48] Q. Yang, Y. Su, C. Chi, C. T. Cherian, K. Huang, V. G. Kravets, F. C. Wang, J. C. Zhang, A. Pratt, A. N. Grigorenko, F. Guinea, A. K. Geim, R. R. Nair, Ultrathin graphene-based membrane with precise molecular sieving and ultrafast solvent permeation. Nat. Mater. 16, 1198–1202 (2017). 29170556 10.1038/nmat5025

[49] L. Huang, J. Chen, T. Gao, M. Zhang, Y. Li, L. Dai, L. Qu, G. Shi, Reduced graphene oxide membranes for ultrafast organic solvent nanofiltration. Adv. Mater. 28, 8669–8674 (2016). 27514930 10.1002/adma.201601606

[50] L. Shen, Q. Shi, S. Zhang, J. Gao, D. C. Cheng, M. Yi, R. Song, L. Wang, J. Jiang, R. Karnik, S. Zhang, Highly porous nanofiber-supported monolayer graphene membranes for ultrafast organic solvent nanofiltration. Sci. Adv. 7, eabg6263 (2021). 34516873 10.1126/sciadv.abg6263 PMC8442935

[51] M. Althuluth, J. P. Overbeek, H. J. van Wees, L. F. Zubeir, W. G. Haije, A. Berrouk, C. J. Peters, M. C. Kroon, Natural gas purification using supported ionic liquid membrane. J. Membr. Sci. 484, 80–86 (2015).

[52] Y. Wu, X. Ma, Y. Li, W. Guan, J. Tong, N. Hu, Theoretical and experimental determination of the number of water molecules breaking the structure of a glycine-based ionic liquid. RSC Adv. 4, 10531–10541 (2014).

[53] S. Zhao, Z. Zhao, Z. Zha, Z. Jiang, Z. Wang, M. D. Guiver, Amine-rich molecular nodule-assembled membrane having 5 angstrom channels for CO_2_/N_2_ separation. Adv. Funct. Mater. 34, 1–9 (2024).

[54] D. Li, R. Wang, T. S. Chung, Fabrication of lab-scale hollow fiber membrane modules with high packing density. Sep. Purif. Technol. 40, 15–30 (2004).

[55] Y. Han, W. Salim, K. K. Chen, D. Wu, W. S. W. Ho, Field trial of spiral-wound facilitated transport membrane module for CO_2_ capture from flue gas. J. Membr. Sci. 575, 242–251 (2019).

[56] W. Chen, Z. Zhang, C. Yang, J. Liu, H. Shen, K. Yang, Z. Wang, PIM-based mixed-matrix membranes containing MOF-801/ionic liquid nanocomposites for enhanced CO_2_ separation performance. J. Membr. Sci. 636, 119581 (2021).

[57] Y. Ban, Z. Li, Y. Li, Y. Peng, H. Jin, W. Jiao, A. Guo, P. Wang, Q. Yang, C. Zhong, W. Yang, Confinement of ionic liquids in nanocages: Tailoring the molecular sieving properties of ZIF-8 for membrane-based CO_2_ capture. Angew. Chem. Int. Ed. Engl. 54, 15483–15487 (2015). 26515558 10.1002/anie.201505508

[58] H. Lin, K. Gong, P. Hykys, D. Chen, W. Ying, Z. Sofer, Y. Yan, Z. Li, X. Peng, Nanoconfined deep eutectic solvent in laminated MXene for efficient CO_2_ separation. Chem. Eng. J. 405, 126961 (2021).

[59] M. Karunakaran, L. F. Villalobos, M. Kumar, R. Shevate, F. H. Akhtar, K. V. Peinemann, Graphene oxide doped ionic liquid ultrathin composite membranes for efficient CO_2_ capture. J. Mater. Chem. A 5, 649–656 (2017).

[60] X. Wan, X. Wang, T. Wan, Y. Yan, Z. Ye, X. Peng, Bio-inspired ferromagnetic graphene oxide/magnetic ionic liquid membrane for highly efficient CO_2_ separation. Appl. Mater. Today 24, 101164 (2021).

[61] W. Guo, S. M. Mahurin, R. R. Unocic, H. Luo, S. Dai, Broadening the gas separation utility of monolayer nanoporous graphene membranes by an ionic liquid gating. Nano Lett. 20, 7995–8000 (2020). 33064492 10.1021/acs.nanolett.0c02860

[62] J. Albo, T. Tsuru, Thin ionic liquid membranes based on inorganic supports with different pore sizes. Ind. Eng. Chem. Res. 53, 8045–8056 (2014).

[63] H. Li, F. Wang, H. Li, B. Sengupta, D. K. Behera, S. Li, M. Yu, Ultra-selective membrane composed of charge-stabilized fixed carrier and amino acid-based ionic liquid mobile carrier for highly efficient carbon capture. Chem. Eng. J. 453, 139780 (2023).

[64] X. M. Zhang, Z. H. Tu, H. Li, L. Li, Y. T. Wu, X. B. Hu, Supported protic-ionic-liquid membranes with facilitated transport mechanism for the selective separation of CO_2_. J. Membr. Sci. 527, 60–67 (2017).

[65] D. K. Behera, F. Wang, B. Sengupta, K. Friedman, S. Li, M. Yu, A facilitated transport membrane composed of amine-containing ionic liquid confined in a GO/CNT network for highly efficient carbon capture. J. Membr. Sci. 712, 123177 (2024).

[66] S. M. Mahurin, J. S. Lee, G. A. Baker, H. Luo, S. Dai, Performance of nitrile-containing anions in task-specific ionic liquids for improved CO_2_/N_2_ separation. J. Membr. Sci. 353, 177–183 (2010).

[67] M. Y. Abdelrahim, C. F. Martins, L. A. Neves, C. Capasso, C. T. Supuran, I. M. Coelhoso, J. G. Crespo, M. Barboiu, Supported ionic liquid membranes immobilized with carbonic anhydrases for CO_2_ transport at high temperatures. J. Membr. Sci. 528, 225–230 (2017).

[68] E. Santos, J. Albo, A. Irabien, Acetate based supported ionic liquid membranes (SILMs) for CO_2_ separation: Influence of the temperature. J. Membr. Sci. 452, 277–283 (2014).

[69] J. Yin, C. Zhang, Y. Yu, T. Hao, H. Wang, X. Ding, J. Meng, Tuning the microstructure of crosslinked Poly(ionic liquid) membranes and gels via a multicomponent reaction for improved CO_2_ capture performance. J. Membr. Sci. 593, 117405 (2020).

[70] J. Zhou, M. M. Mok, M. G. Cowan, W. M. McDanel, T. K. Carlisle, D. L. Gin, R. D. Noble, High-permeance room-temperature ionic-liquid-based membranes for CO_2_/N_2_ separation. Ind. Eng. Chem. Res. 53, 20064–20067 (2014).

[71] J. Y. Lim, J. K. Kim, C. S. Lee, J. M. Lee, J. H. Kim, Hybrid membranes of nanostructrual copolymer and ionic liquid for carbon dioxide capture. Chem. Eng. J. 322, 254–262 (2017).

[72] S. Janakiram, L. Ansaloni, S. A. Jin, X. Yu, Z. Dai, R. J. Spontak, L. Deng, Humidity-responsive molecular gate-opening mechanism for gas separation in ultraselective nanocellulose/IL hybrid membranes. Green Chem. 22, 3546–3557 (2020).

[73] V. A. Kusuma, M. K. Macala, J. Liu, A. M. Marti, R. J. Hirsch, L. J. Hill, D. Hopkinson, Ionic liquid compatibility in polyethylene oxide/siloxane ion gel membranes. J. Membr. Sci. 545, 292–300 (2018).

[74] M. Sandru, T. J. Kim, W. Capala, M. Huijbers, M. B. Hägg, Pilot scale testing of polymeric membranes for CO_2_ capture from coal fired power plants. Energy Procedia 37, 6473–6480 (2013).

[75] X. Xu, J. Dong, X. Xiao, X. Zhao, Q. Zhang, Constructing thin and cross-linked polyimide membranes by interfacial reaction for efficient CO_2_ separation. ACS Sustain. Chem. Eng. 9, 5546–5556 (2021).

[76] J. M. P. Scofield, P. A. Gurr, J. Kim, Q. Fu, S. E. Kentish, G. G. Qiao, Development of novel fluorinated additives for high performance CO_2_ separation thin-film composite membranes. J. Membr. Sci. 499, 191–200 (2016).

[77] L. S. White, K. D. Amo, T. Wu, T. C. Merkel, Extended field trials of Polaris sweep modules for carbon capture. J. Membr. Sci. 542, 217–225 (2017).

[78] P. Li, Z. Wang, W. Li, Y. Liu, J. Wang, S. Wang, High-performance multilayer composite membranes with mussel-inspired polydopamine as a versatile molecular bridge for CO_2_ separation. ACS Appl. Mater. Interfaces 7, 15481–15493 (2015). 26121208 10.1021/acsami.5b03786

[79] S. Li, Z. Wang, X. Yu, J. Wang, S. Wang, High-performance membranes with multi-permselectivity for CO_2_ separation. Adv. Mater. 24, 3196–3200 (2012). 22605654 10.1002/adma.201200638

[80] K. J. Howe, M. M. Clark, Fouling of microfiltration and ultrafiltration membranes by natural waters. Environ. Sci. Technol. 36, 3571–3576 (2002). 12214651 10.1021/es025587r

