# Supplementary material for: Scalable nanoconfined ionic liquid membranes with ultrapermeance and ultraselectivity for efficient CO2 capture
Source: Sci Adv. 2026 Jan 7;12(2):eaea1329. doi: 10.1126/sciadv.aea1329 (PMC12778053; doi:10.1126/sciadv.aea1329)
Supplement: Supplementary file 1 — Supplementary Text Figs. S1 to S19 Tables S1 to S6 References [file sciadv.aea1329_sm.pdf]

Science Advances
AAAS

Supplementary Materials for

Scalable nanoconfined ionic liquid membranes with ultrapermeance and ultraselectivity for efficient CO_2 capture

Fan Wang *et al.*

Corresponding author: Miao Yu, myu9@buffalo.edu

Sci. Adv. **12**, eaea1329 (2026)
DOI: 10.1126/sciadv.aea1329

This PDF file includes:

Supplementary Text
Figs. S1 to S19
Tables S1 to S6
References

PEG Rejection and Effective Pore Size Characterization

Effective pore size and pore size distribution of NCIL membranes were determined by the rejection of a series of PEGs/PEOs (10 kDa, 100 kDa, 300 kDa, 1000 kDa, 5000 kDa). The hydrodynamic diameter (d_s) of PEG molecules (*80*) was calculated based on Eq. S1 and MW is the molecular weight of PEG. Effective pore radius (r_p) was determined by modified Ferry's equation (Eq. S2) for 90% Rejection (*R*) of PEG (*43*), where r_s is the hydrodynamic radius of the PEG/PEO solute:

$$d_s\ (nm) = 0.09 \times \ \mathrm{MW}^{0.44} \qquad \text{(Eq. S1)}$$

$$R = 1 - \left(1 - \frac{r_s}{r_p}\right)^2 - \left(1 - \frac{r_s}{r_p}\right)^4 \qquad \text{(Eq. S2)}$$

Theoretical Capillary/Breakthrough Pressure Calculation

The theoretical capillary breakthrough pressure of the CNT mesh was calculated to estimate effectiveness of nanoconfinement, following the simplified Young-Laplace equation (*51*). As indicated in Equation (2), the γ is surface tension of ionic liquid [Emim][Gly] of 53.5 mN·m^{-2} (*52*); θ is the contact angle between the ionic liquid [Emim][Gly] and the pore surface of 45° (Fig. S7); r_p is effective pore radius of CNT mesh, with minimum value of 10 nm as demonstrated in Fig. 2B. Given all the parameters mentioned above, the theoretical breakthrough pressure ranges from 25 bar to 152 bar.

Single and Mixed Gas Permeation Measurement

For small area flat sheet membrane, the gas permeance P_i of component i was calculated by:

$$P_i = \frac{J_i}{A \cdot \Delta P_i} \qquad \text{(Eq. S3)}$$

$$\Delta P_i = P_{f,i} - P_{p,i} \qquad \text{(Eq. S4)}$$

Where A is the effective membrane area, J_i is molar flow rate of gas component i through the membrane, ΔP_i represents partial pressure difference of gas component i between membrane feed side and permeate side. The gas separation selectivity α_ij was calculated by:

$$\alpha_\mathrm{ij} = \frac{P_\mathrm{i}}{P_\mathrm{j}} \qquad \text{(Eq. S5)}$$

For gas mixture permeation measurement of scale-up (75 cm^2) hollow fiber NCIL membrane, the system setup was similar to that for flat sheet membrane. The gas permeance P_i of component i was calculated:

$$P_\mathrm{i} = \frac{J_\mathrm{i}}{A \cdot \Delta P_\mathrm{i,ln}} \qquad \text{(Eq. S6)}$$

$$\Delta P_\mathrm{i,ln} = \left(\frac{(P_\mathrm{f,i} - P_\mathrm{p,i}) - (P_\mathrm{r,i} - P_\mathrm{p,i})}{\ln{(P_\mathrm{f,i} - P_\mathrm{r,i})}}\right) - P_\mathrm{p,i} \qquad \text{(Eq. S7)}$$

Where $\Delta P_\mathrm{i,ln}$ is log-mean pressure drop through the hollow fiber, simplified model was applied to calculate driving force of species i. $P_\mathrm{f,i}$, $P_\mathrm{r,i}$, $P_\mathrm{p,i}$ represent partial pressure of gas component i in feed, retentate, permeate side of membrane. The selectivity α_ij was calculated based on Eq. S5.

The CO_2 capture rate and dry-basis purity of hollow fiber membrane were calculated by:

$$\mathrm{CO_2\ capture\ rate} = \frac{F_\mathrm{p,\,CO_2}}{F_\mathrm{f,\,CO_2}} \times 100\% \qquad \text{(Eq. S8)}$$

$$\mathrm{CO_2\ drybasis\ purity} = \frac{F_\mathrm{p,\,CO_2}}{F_\mathrm{p,\,CO_2} + F_\mathrm{p,\,N_2}} \times 100\% \qquad \text{(Eq. S9)}$$

Where $F_\mathrm{p,\,CO_2}$, $F_\mathrm{p,\,N_2}$ are the flow rate of CO_2 and N_2 in permeate side of hollow fiber membrane, respectively. $F_\mathrm{f,\,CO_2}$ is the flow rate of CO_2 in feed side of hollow fiber membrane.

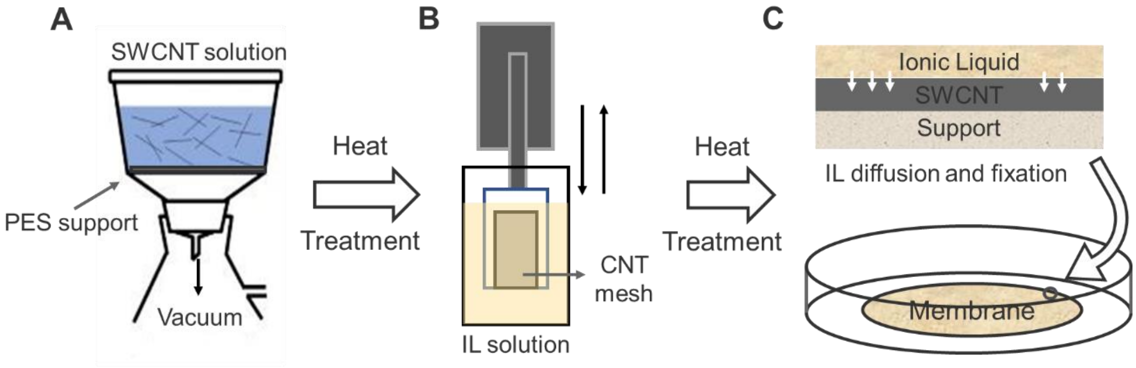

Fig. S1. Schematic of NCIL flat sheet membrane fabrication. (**A**) Vacuum filtration for CNT mesh coated PES membrane preparation; (**B**) Dip coating for IL loading onto CNT mesh; (**C**) Demonstration of IL impregnation into CNT mesh for NCIL membranes formation.

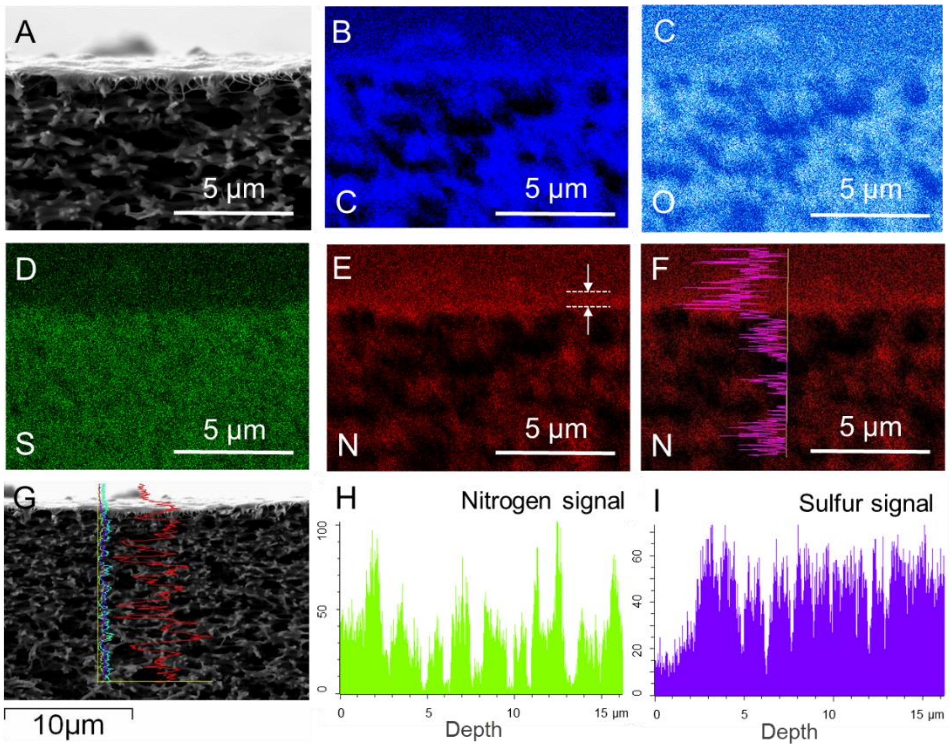

Fig. S2. SEM and EDS images of nanoconfined NCIL membrane. (**A**) cross-section image of membrane; (**B**)-(**F**) EDS elemental mapping for C, O, S and N elements; (**E**) EDS elemental mapping and line scanning patten for N element; (**G**)-(**I**) EDS line scanning patterns and N, S elements density distribution in different part of membrane. EDS results indicate that ionic liquid [Emim][Gly] is concentrated on the top of support to form continuous thin membrane layer, but IL distributed within cannot form a continuous layer even though IL density is similar.

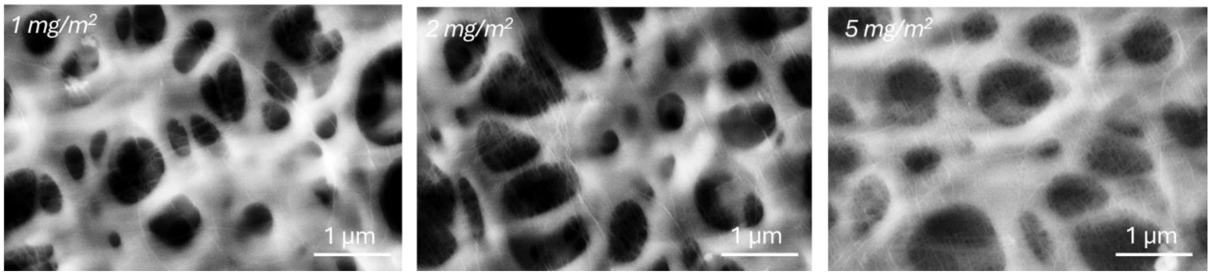

Fig. S3. Surface SEM images of CNT mesh coated onto PES support. CNT loading density ranges from 1 mg m^{-2} to 5 mg m^{-2}, exhibiting the gradual formation of dense CNT film .

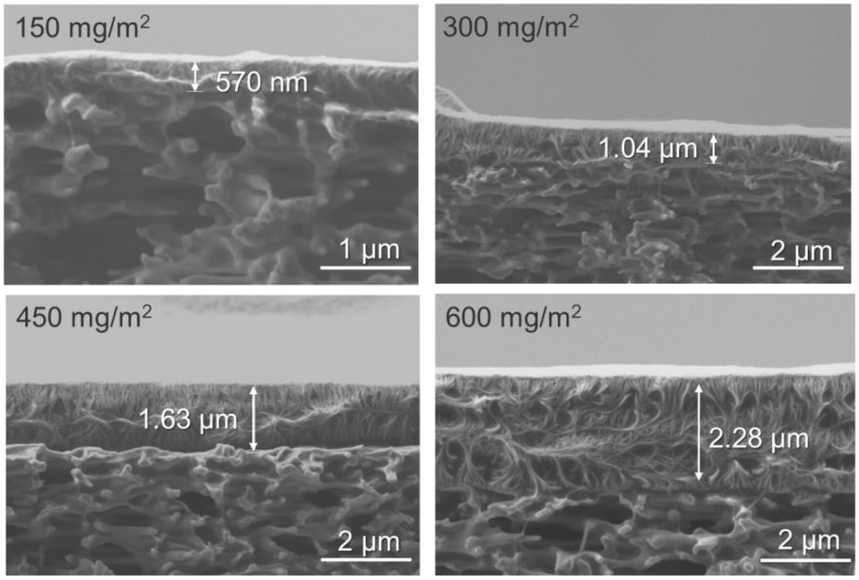

Fig. S4. Cross-section SEM images of CNT mesh coated onto PES support. CNT loading density ranges from 150 mg m^{-2} to 600 mg m^{-2}. The thickness of CNT mesh increased linearly with CNT loading density when CNT mesh thickness is higher than 500 nm.

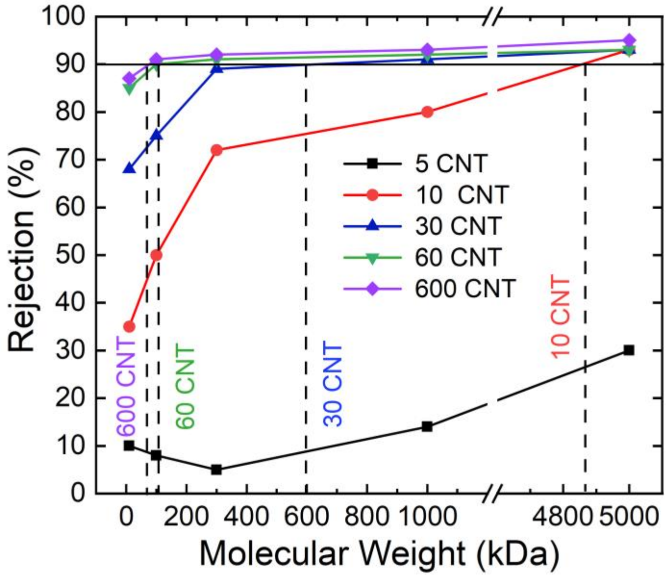

Fig. S5. The Rejection experiment of PEOs for CNT mesh on PES support. CNT loading density ranges from 10 mg m^{-2} to 600 mg m^{-2}. The rejection test of CNT mesh/PES membrane was conducted under 0.5 bar feed side pressure.

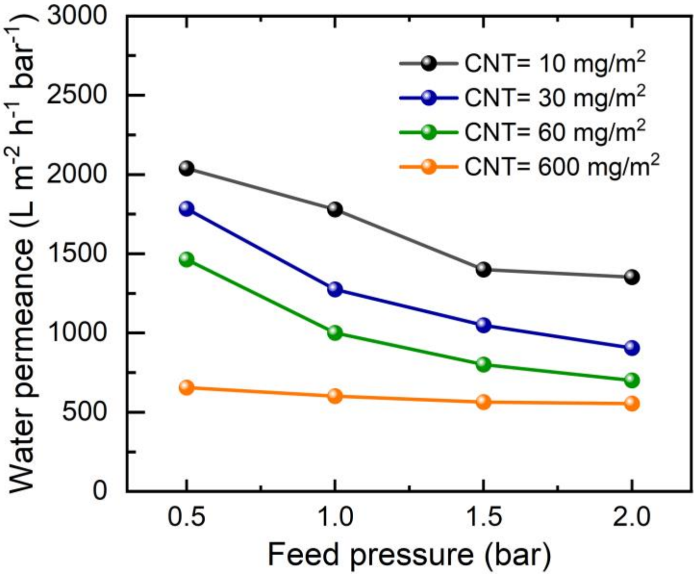

Fig. S6. Water permeance of CNT mesh coated PES membrane under different feed pressure. The figure showed compressibility of CNT mesh under pressure. Thicker CNT membrane can handle higher pressure without obvious structure deformation.

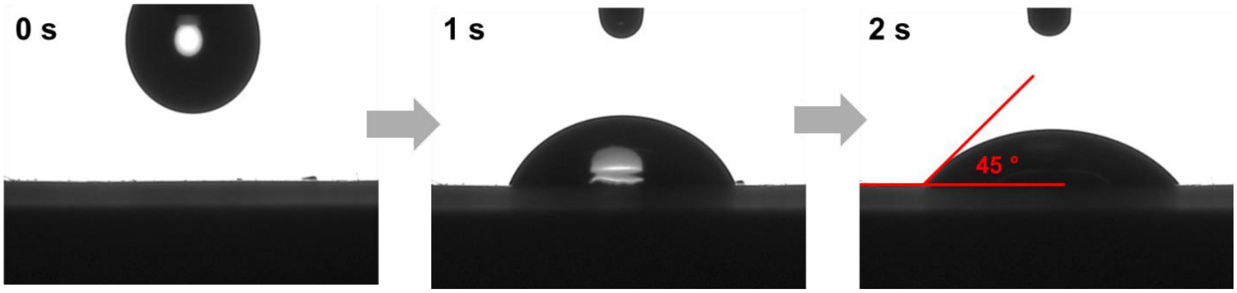

Fig. S7. Contact angle of ionic liquid [Emim][Gly] onto CNT mesh coated PES membrane. The CNT loading density onto PES was controlled at 60 mg m^{-2}.

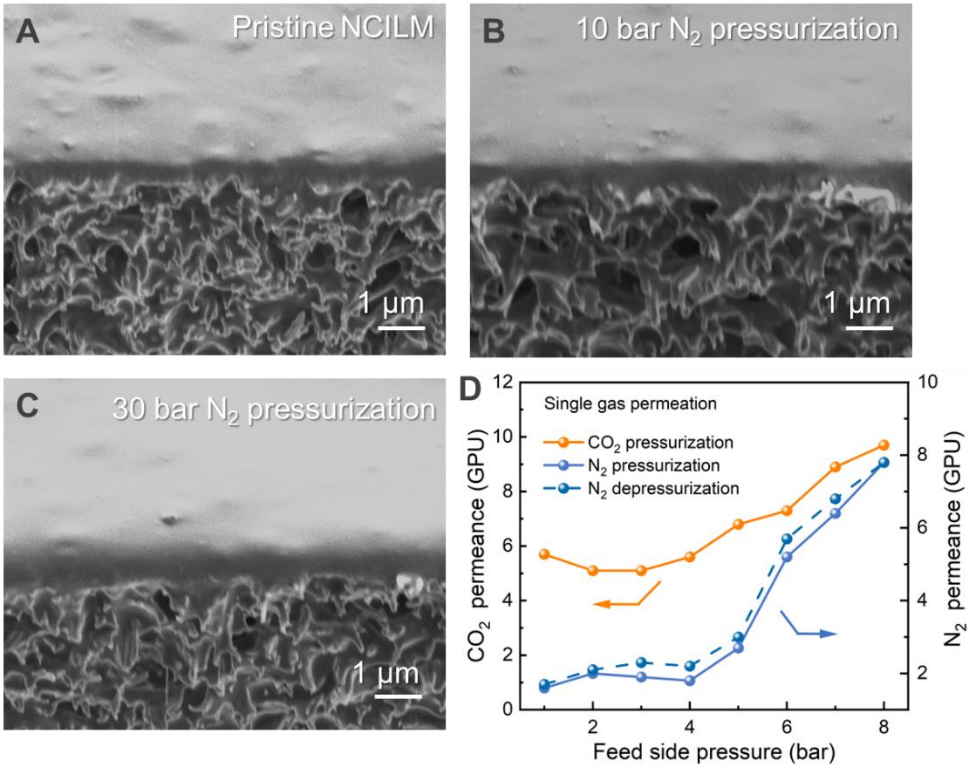

Fig. S8. Demonstration of effective nanoconfinement of ionic liquid within NCIL membranes. (A)-(C) Cross-section SEM images of NCIL membrane before and after high pressurization test; (**D**) Reversible gas permeation test after pressurization and depressurizations process.

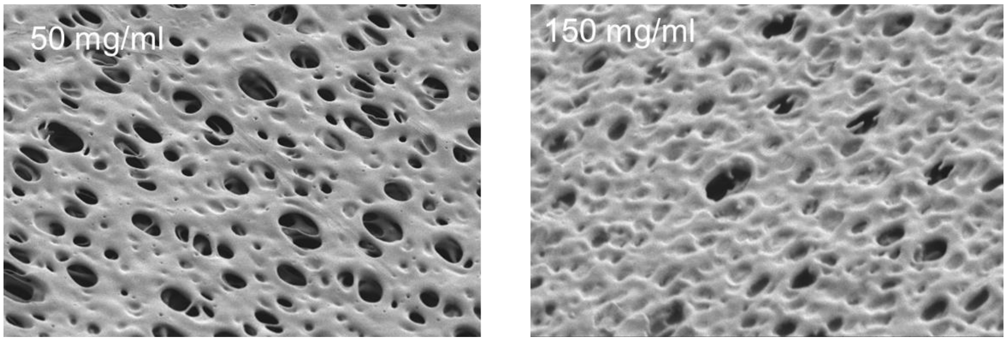

Fig. S9. Surface SEM images of IL-coated PES support with different IL loading amount.

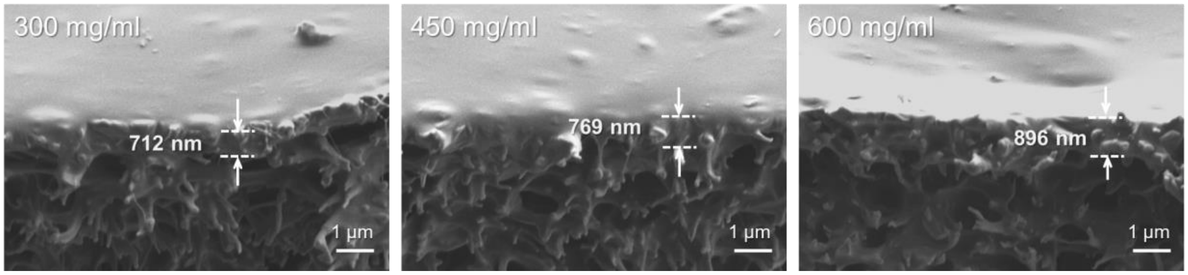

Fig. S10. Cross-section SEM images of NCIL membranes. The membranes were prepared with different concentrations of IL coating solutions ranging from 50 to 600 mg mL^{-1} with CNT loading density of 60 mg m^{-2}.

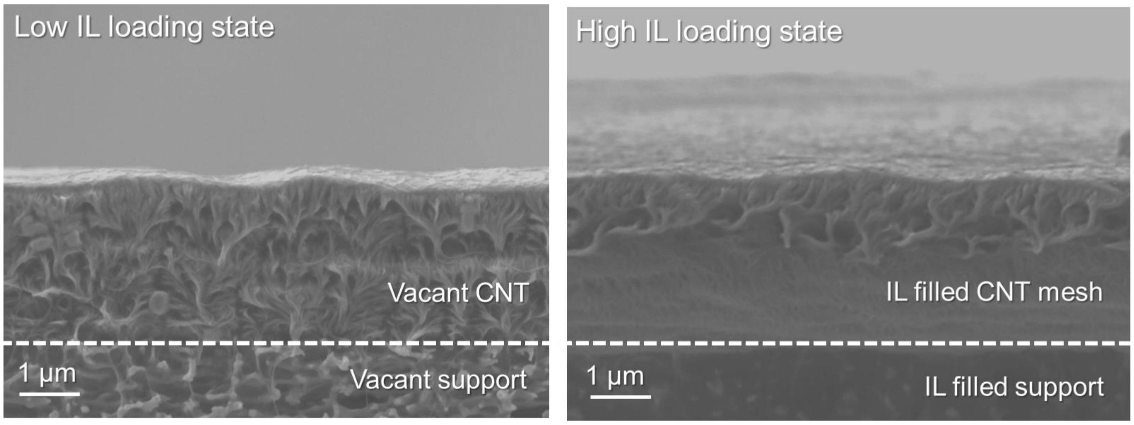

Fig. S11. Cross-section SEM images of NCIL membranes. The membranes were prepared with different concentrations of IL coating solutions ranging from 150 to 450 mg mL^{-1} with CNT loading density of 600 mg m^{-2}.

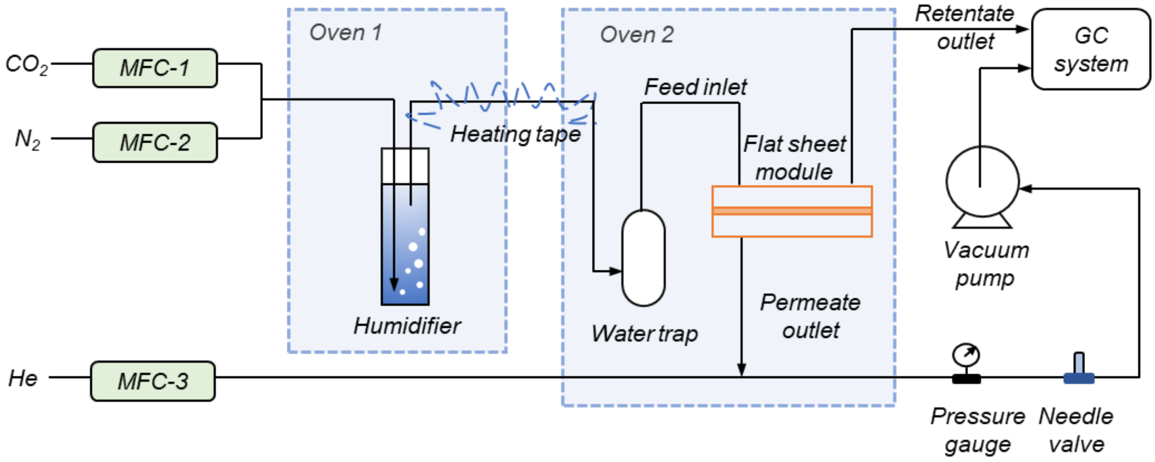

Fig. S12. Schematic of gas separation performance evaluation system (flat sheet membrane). Dry CO_2/N_2 gas mixture was introduced into humidifier to make up feed gas with different water vapor composition. Mass flow controller in the retentate side of membrane was used to adjust membrane feed side pressure via controlling the retentate side gas flow rate. Permeate side pressure was controlled by needle valve between membrane permeate outlet and vacuum pump, with the aid of Helium as carrier gas.

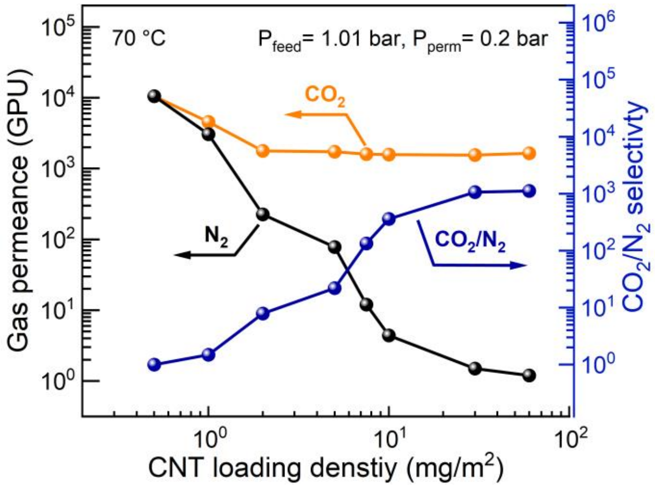

Fig. S13. Gas separation performance of the NCIL membranes as a function of CNT loading density with IL solution of 150 mg mL^{-1} for dip-coating. The resulting NCIL membranes were tested using simulated flue gas (4.2% CO_2, saturated H_2O vapor, and balanced N_2) under 1.01 bar feed pressure (absolute) and 0.2 bar (absolute) permeate side pressure at 70°C.

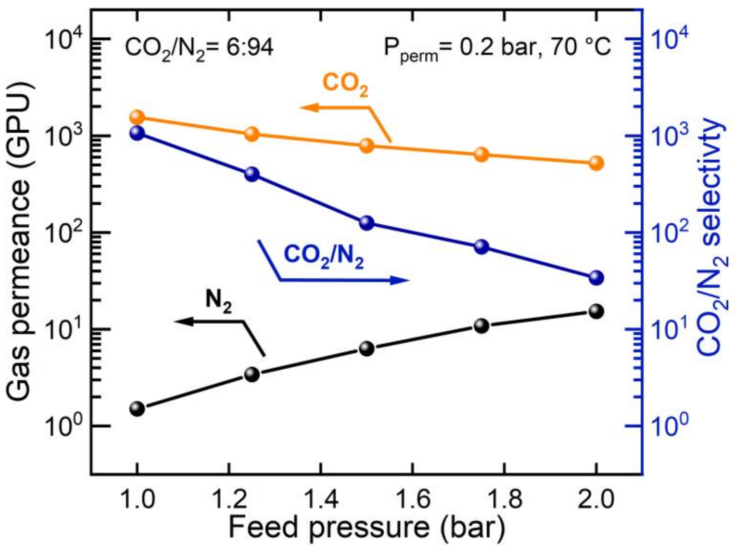

Fig. S14. Gas separation performance of NCIL-C60/IL150 membrane as a function of feed pressure in feed side. NCIL membranes were tested using simulated flue gas composed of 4.2% CO_2, saturated H_2O vapor, and balanced N_2 under 0.2 bar (absolute) permeate side pressure, 70°C.

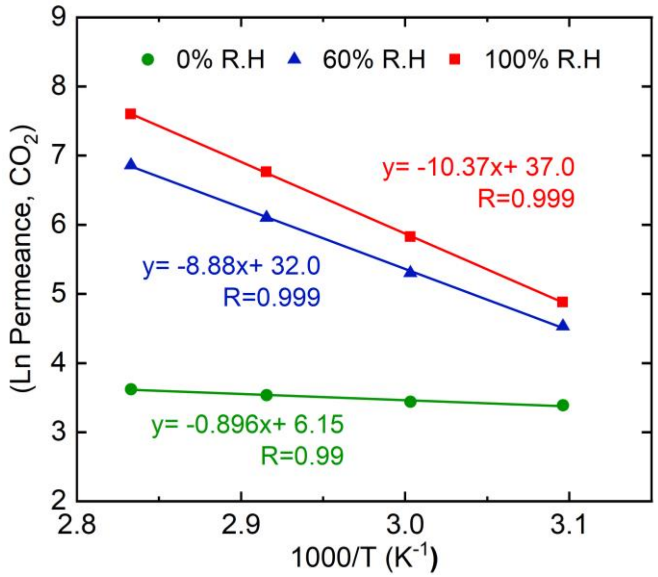

Fig. S15. The ln (Permeance, CO_2) – 1/T plot for CO_2 permeation activation energy of NCIL membrane under different relative humidity (R.H). The feed gas composed of 10.5 vol% CO_2, 0~31.2 vol% H_2O and balanced N_2 was continuously measured at 0.2 bar permeate side pressure, 70°C. Feed side pressure was controlled at 1.01 bar.

The activation energy of CO_2 permeation, E_{a} is calculated from Arrhenius equation:

$$P_{\mathrm{CO_2}}(T) = A_0 \exp\left(\frac{-E_{\mathrm{a}}}{RT}\right)$$

where A_0 is the pre-factor of Arrhenius equation, $P_{\mathrm{CO_2}}(T)$ is CO_2 permeance of NCIL membrane under different temperature, and R is the ideal gas constant.

The plot shows the CO_2 permeation activation energy decreases (from 86.23 kJ/mol to 73.86 kJ/mol and 7.29 kJ/mol) with decreased relative humidity (from 100% to 60% and 0%) in the feed gas. Specifically, CO_2 permeation activation energy is much lower for dry feed gas, probably because CO_2 transport through membrane only follow solution-diffusion mechanism without water vapor. The high activation energy under wet conditions reveals that high temperature favors CO_2 transport with the presence of water.

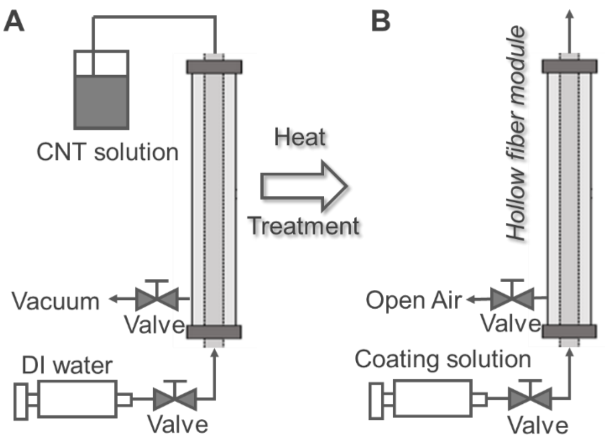

Fig. 16. Scaled-up NCIL hollow fiber membrane fabrication. (**A**) Vacuum filtration of CNT onto inner surface of PES hollow fiber support; (**B**) Dip-coating of IL solution through CNT mesh coated PES support (see detailed fabrication procedure in Materials and Methods).

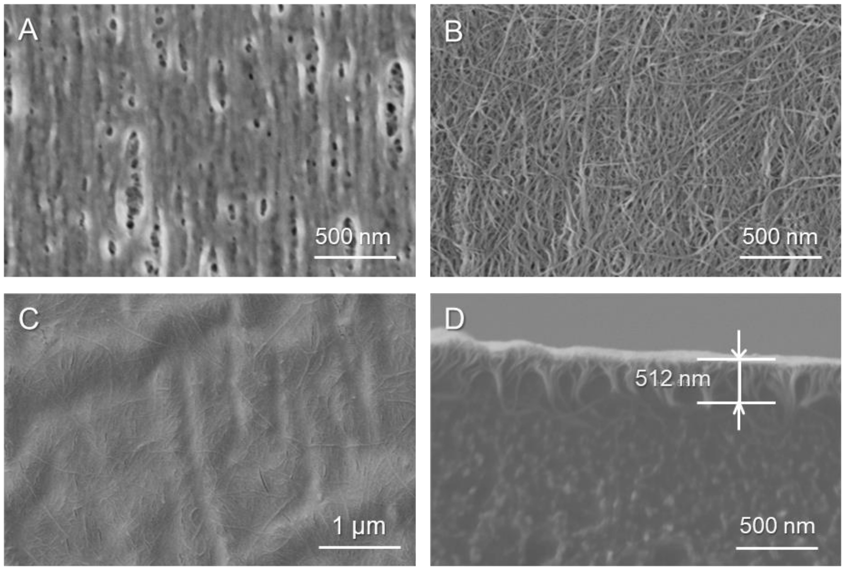

Fig. S17. SEM images of scaled-up hollow fiber NCIL membranes. (**A**) 75 cm^2 hollow fiber PES support (300 kDa); (**B**) CNT loaded hollow fiber support (60 mg m^{-2} CNT loading density); (**C**) NCIL hollow fiber membrane (450 mg mL^{-1} IL concentration in coating solution). (**D**) Cross-section SEM image of NCIL membrane.

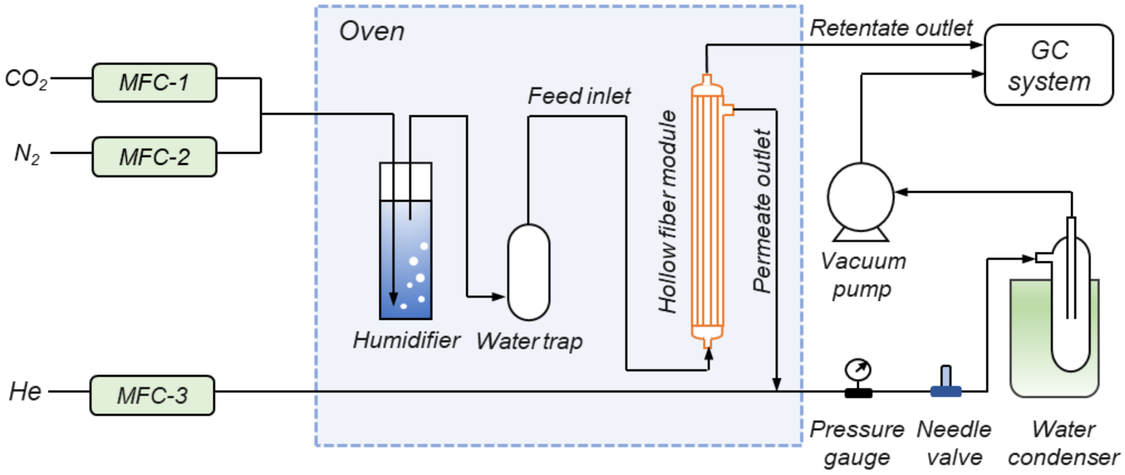

Fig. S18. Schematic of CO_2/N_2 mixed gas separation performance evaluation system for scaled-up 75 cm^2 hollow fiber NCIL membrane. The feed gas was introduced from bottom inlet of hollow fiber (feed side) to the top outlet (Retentate side), and vacuum pressure (permeate side) was applied onto the shell side of hollow fiber module to provide the driving force.

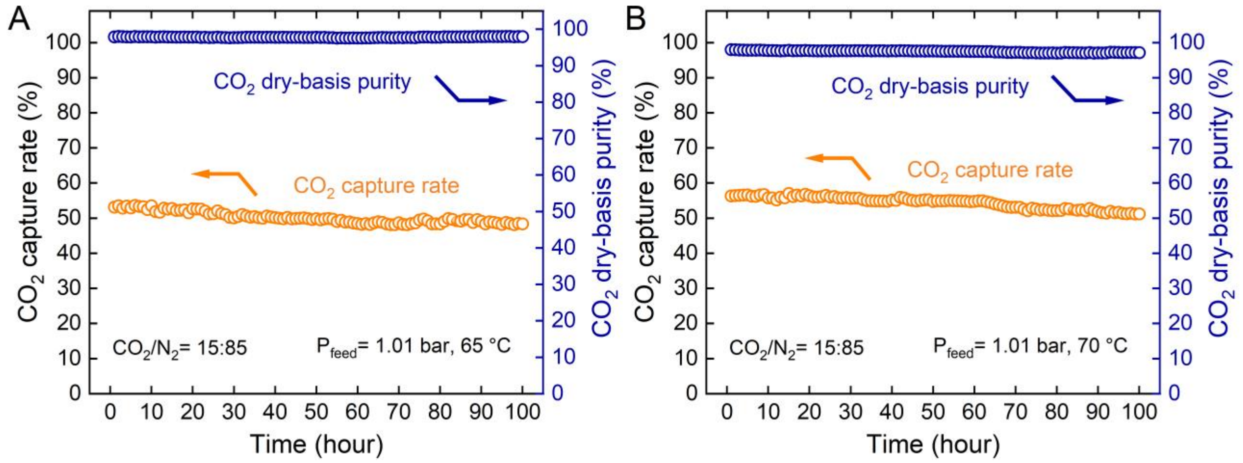

Fig. S19. Long-term stability results of scaled-up 75 cm^2 NCIL hollow fiber membranes for CO_2 capture from simulated coal-fire flue gas. (**A**) feed gas composed of 11.3 vol% CO_2, 25 vol% H_2O and balanced N_2 at 65°C and (**B**) feed gas composed of 10.5 vol% CO_2, 31.2 vol% H_2O and balanced N_2 at 70°C. Unless otherwise specified, the feed side pressure and permeate side pressure were controlled at 1.01 bar (absolute pressure) and 0.15 bar (absolute pressure). The mixture gas introduced before humidifier was controlled at 200 sccm.

Table S1. Comparison of porosity over tortuosity factor (ε/τ) of nano-structure (NS) membranes.

Description	Permeance (L m^{-2} s^{-1} bar^{-1})	Effective pore size (δ, nm)	Thickness (nm)	ε/τ	Reference
PA	0.010	0.83	9	6.57E-03	[45]
PIM	0.016	1.4	88	4.03E-02	[46]
Mesoporous Phenolics	0.027	5	250	1.51E-02	[47]
	0.014	3.5	500	3.91E-02	
GO	0.003	0.9	8	1.37E-03	[48]
rGO	0.020	1.5	25	1.27E-02	[49]
Graphene	0.067	1.52	1	1.63E-03	[50]
CNT	2.200	59.3	38	2.42E-03	This work
	0.825	25.7	114	1.45E-02	
	0.599	11.8	228	1.00E-01	
	0.118	9.8	2280	6.77E-02	

* Permeation test of CNT mesh coated PES membrane was conducted under 0.5 bar feed side pressure.

Table S2. CO_2/N_2 mixed gas separation performance of dip-coated IL/PES support.

PES pore size (nm)	CO_2 permeance (GPU)	N_2 permeance (GPU)	CO_2/N_2 selectivity
450	>10000		1.01
100	10010	9415	1.06
30	3378	1972	1.76

* IL concentration in coating solution was maintained at 200 mg mL^{-1} and IL dip-coated PES supports were tested using CO_2/N_2 mixture (1: 1 ratio) under ambient pressure, 25°C.

Table S3. Comparison of CO_2 separation performance of nanoconfined IL membranes in literatures.

Confinement type	Confinement materials	Confined pore or channel size (nm)	Ionic liquid	Temperature (K)	CO_2 permeance (GPU)	CO_2/N_2 selectivity	Membrane thickness	Reference
Nanocage	MOF-801	0.7	[Bmim][NTf_2]	308	377	29	25 μm	[56]
	ZIF-8*	0.6	[Bmim][Tf_2N]	303	16	116	30 μm	[57]
Nanochannel	$Ti_3C_2T_x$*	1.5	1ChCl-4EG	293	26	319	2 μm	[58]
	GO	1.9	[Bmim][BF_4]	323	68	382	1 μm	[37]
	GO	1.1	[Emim][Ac]	298	37	130	400 nm	[59]
	GO*	1.3	[$P_{6,6,6,14}$][$FeCl_4$]	298	83	180	45 nm	[60]
	WS_2	1.2	[Bmim][BF_4]	298	50	153	150 nm	[38]
	MoS_2*	1.2	[Bmim][BF_4]	298	48	131	740 nm	[39]
Nanopore	Nano-porous Graphene	1 .0	[Bmim][BF_4]	295	4000	32	400 nm	[61]
	TiO_2	2.5	[Emim][Ac]	298	67.5	34	780 nm	[62]
	CNT*	9.8	[Emim][Gly]	343	1654	1132	560 nm	This work

* Membrane separation performance was evaluated using CO_2 and N_2 gas mixture. 1 GPU: gas permeation unit; 1 GPU= 3.35×10^{-10} mol $(m^2\cdot s\cdot Pa)^{-1}$.

Table S4. Comparison of CO_2 separation performance of IL-based membranes in reported literatures.

Membrane type	Support	Ionic liquid	Temperature (K)	CO_2 permeance (GPU)	CO_2/N_2 selectivity	Membrane thickness	Reference
SILMs	PES	[DMAPAH][TFA]	303	13	90	120 μm	[64]
		[Emim][B(CN)$_4$]	298	34	53	60 μm	[66]
	PVDF	[C$_4$mim][NTf$_2$]	373	5.8	36	125 μm	[67]
		[Vbtma][Ac]	298	8.8	39	125 μm	[68]
PILMs		Poly[Emim][C(CN)$_3$]/[Emim][C(CN)$_3$]	293	3.6	64	120 μm	[69]
		Poly[VHLM][Tf$_2$N]/[Emim][Tf$_2$N]	298	6400	25	100 nm	[70]
ILCMs		SBS-g-POEM/[Emim][DCA]	308	4.6	25	100 μm	[71]
		NF cellulose/[Emim][Ac]*	308	5.5	370	60 μm	[72]
		PEO/[P$_{1444}$][Tf$_2$N]	313	1.3	24	230 μm	[73]
FTILMs*		CNT-PSS/[Emim][Gly]	343	1285	752	235 nm	[63]
		CNT-GO/[Emim][Gly]		1600	400	230 nm	[65]
		CNT/[Emim][Gly]		1654	1132	560 nm	This work

† Membrane type abbreviation: Supported ionic liquid membranes (SILMs), Poly(ionic liquid) membranes (PILMs), Ionic liquid composite membranes (ILCMs), Facilitated transport ionic liquid membranes (FTILMs).

* Membrane separation performance was evaluated using CO_2 and N_2 gas mixture. 1 GPU: gas permeation unit; 1 GPU= 3.35×10^{-10} mol $(m^2\cdot s\cdot Pa)^{-1}$.

Table S5. Comparison of CO_2 separation performance of conventional thin film polymeric and facilitated transport membranes in the reported literatures.

Membrane type†	Membrane description	Temperature (K)	CO_2 permeance (GPU)	CO_2/N_2 selectivity	Membrane thickness	Reference
PMs	PI	308	300	36	103 nm	[75]
	PEG-b-PPFPA	308	1830	17	870 nm	[76]
	Polaris 2nd Gen*	303	1670	50	100 nm	[77]
FTMs*	PVAm/K-Gly	330	903	173	350 nm	[19]
	PVAm (Pilot)	318	260	300	1.2 µm	[74]
	PVAm/PDA	298	1887	83	165 nm	[78]
	TMC/DNMDAm/DGBAmE	295	1613	138	500 nm	[79]
	PNVF-co-Vam/PZEA-Sar (Pilot)	340	1450	185	170 nm	[55]
LFTMs*	CNT/PSS/PEI	353	820	460	230 nm	[28]
	GO/EDA	348	660	572	28 nm	[29]
	CNT/PSS/TEPA	353	1280	450	220 nm	[34]
	CNT/[Emim][Gly]	343	1654	1132	560 nm	This work

† Membrane type abbreviation: Polymeric membranes (PMs), Facilitated transport membranes (FTMs), Liquid-based facilitated transport membranes (LFTMs).

* Membrane separation performance was evaluated using CO_2 and N_2 gas mixture. 1 GPU: gas permeation unit; 1 GPU= 3.35×10^{-10} mol $(m^2\cdot s\cdot Pa)^{-1}$.

Table S6. Gas separation performance of scaled-up 75 cm^2 NCIL hollow fiber membranes.

**Dry feed flow rate (sccm)**	**CO_2 permeance (GPU)**	**N_2 permeance (GPU)**	**CO_2/N_2 selectivity**	**CO_2 capture rate (%)**	**CO_2 dry-basis purity (%)**
100	835	0.78	1068	59.1	94.6
150	1167	0.83	1415	56.7	96.0
200	1552	0.86	1803	55.2	96.8
250	1884	0.9	2092	53.1	97.2
300	1986	0.85	2344	49.8	97.6
**Permeate side pressure (bar)**	**CO_2 permeance (GPU)**	**N_2 permeance (GPU)**	**CO_2/N_2 selectivity**	**CO_2 capture rate (%)**	**CO_2 dry-basis purity (%)**
0.1	1344	1.18	1143	54.3	97.0
0.15	1986	0.85	2344	49.8	97.6
0.20	2068	1.01	2053	38.4	96.5
0.25	1613	1.08	1490	25.7	94.6
0.30	311	0.98	318	7.14	84.8

* The separation performance of 75 cm^2 NCIL hollow fiber membranes were evaluated under 1.01 bar feed pressure and 0.2 bar permeate side pressure (70°C), using simulated NGCC flue gas composed of 4.2 vol% CO_2, 31.2 vol% H_2O and balanced N_2.

REFERENCES

1. M. R. Raupach, C. Le Quéré, G. P. Peters, J. G. Canadell, Anthropogenic CO_2 emissions. *Nat. Clim. Change* **3**, 603–604 (2013).

2. S. I. Seneviratne, M. G. Donat, A. J. Pitman, R. Knutti, R. L. Wilby, Allowable CO_2 emissions based on regional and impact-related climate targets. *Nature* **529**, 477–483 (2016).

3. M. E. Boot-Handford, J. C. Abanades, E. J. Anthony, M. J. Blunt, S. Brandani, N. Mac Dowell, J. R. Fernández, M. C. Ferrari, R. Gross, J. P. Hallett, R. S. Haszeldine, P. Heptonstall, A. Lyngfelt, Z. Makuch, E. Mangano, R. T. J. Porter, M. Pourkashanian, G. T. Rochelle, N. Shah, J. G. Yao, P. S. Fennell, Carbon capture and storage update. *Energy Environ. Sci.* **7**, 130–189 (2014).

4. S. Chu, Carbon capture and sequestration. *Science* **325**, 1599 (2009).

5. Y. Li, X. Yang, E. Du, Y. Liu, S. Zhang, C. Yang, N. Zhang, C. Liu, A review on carbon emission accounting approaches for the electricity power industry. *Appl. Energy* **359**, 122681 (2024).

6. Q. Zhou, A. Manuilova, J. Koiwanit, L. Piewkhaow, M. Wilson, C. W. Chan, P. Tontiwachwuthikul, A comparative of life cycle assessment of post-combustion, pre-combustion and oxy-fuel CO_2 capture. *Energy Procedia* **63**, 7452–7458 (2014).

7. US Energy Information Administration, "Electric power monthly" (2025); https://www.eia.gov/electricity/monthly/.

8. R. L. Siegelman, P. J. Milner, E. J. Kim, S. C. Weston, J. R. Long, Challenges and opportunities for adsorption-based CO_2 capture from natural gas combined cycle emissions. *Energy Environ. Sci.* **12**, 2161–2173 (2019).

9. M.-B. Hägg, A. Lindbråthen, CO_2 capture from natural gas fired power plants by using membrane technology. *Ind. Eng. Chem. Res.* **44**, 7668–7675 (2005).

10. R. W. Baker, B. T. Low, Gas separation membrane materials: A perspective. *Macromolecules* **47**, 6999–7013 (2014).

11. D. L. Gin, R. D. Noble, Designing the next generation of chemical separation membranes. *Science* **332**, 674–676 (2011).

12. R. Hou, C. Fong, B. D. Freeman, M. R. Hill, Z. Xie, Current status and advances in membrane technology for carbon capture. *Sep. Purif. Technol.* **300**, 121863 (2022).

13. Y. Ji, M. Zhang, K. Guan, J. Zhao, G. Liu, W. Jin, High-performance CO_2 capture through polymer-based ultrathin membranes. *Adv. Funct. Mater.* **29**, 1–9 (2019).

14. S. Wang, X. Li, H. Wu, Z. Tian, Q. Xin, G. He, D. Peng, S. Chen, Y. Yin, Z. Jiang, M. D. Guiver, Advances in high permeability polymer-based membrane materials for CO_2 separations. *Energy Environ. Sci.* **9**, 1863–1890 (2016).

15. Z. Dai, L. Ansaloni, L. Deng, Recent advances in multi-layer composite polymeric membranes for CO_2 separation: A review. *Green Energy Environ.* **1**, 102–128 (2016).

16. B. Comesaña-Gándara, J. Chen, C. G. Bezzu, M. Carta, I. Rose, M.-C. Ferrari, E. Esposito, A. Fuoco, J. C. Jansen, N. B. McKeown, Redefining the Robeson upper bounds for CO_2/CH_4 and CO_2/N_2 separations using a series of ultrapermeable benzotriptycene-based polymers of intrinsic microporosity. *Energy Environ. Sci.* **12**, 2733–2740 (2019).

17. L. M. Robeson, The upper bound revisited. *J. Membr. Sci.* **320**, 390–400 (2008).

18. M. Sandru, M. Prache, T. Macron, L. Căta, M. G. Ahunbay, M.-B. Hägg, G. Maurin, M. Barboiu, Rubbery organic frameworks (ROFs) toward ultrapermeable CO_2-selective membranes. *Sci. Adv.* **10**, eadq5024 (2025).

19. Y. Chen, L. Zhao, B. Wang, P. Dutta, W. S. Winston Ho, Amine-containing polymer/zeolite Y composite membranes for CO_2/N_2 separation. *J. Membr. Sci.* **497**, 21–28 (2016).

20. J. G. Wijmans, R. W. Baker, The solution-diffusion model: A review. *J. Membr. Sci.* **107**, 1–21 (1995).

21. Z. Tong, W. S. W. Ho, Facilitated transport membranes for CO_2 separation and capture. *Sep. Sci. Technol.* **52**, 156–167 (2017).

22. T.-Y. Chen, X. Deng, L.-C. Lin, W. S. W. Ho, New sterically hindered polyvinylamine-containing membranes for CO_2 capture from flue gas. *J. Membr. Sci.* **645**, 120195 (2022).

23. M. Sandru, E. M. Sandru, W. F. Ingram, J. Deng, P. M. Stenstad, L. Deng, R. J. Spontak, An integrated materials approach to ultrapermeable and ultraselective CO_2 polymer membranes. *Science* **376**, 90–94 (2022).

24. Y. Han, W. S. W. Ho, Mitigated carrier saturation of facilitated transport membranes for decarbonizing dilute CO_2 sources: An experimental and techno-economic study. *J. Membr. Sci. Lett.* **2**, 100014 (2022).

25. B. Belaissaoui, D. Willson, E. Favre, Membrane gas separations and post-combustion carbon dioxide capture: Parametric sensitivity and process integration strategies. *Chem. Eng. J.* **211-212**, 122–132 (2012).

26. A. Chamoun-Farah, A. N. Keller, M. Y. Balogun, L. M. Cañada, J. F. Brennecke, B. D. Freeman, Amine functionalized supported ionic liquid membranes (SILMs) for CO_2/N_2 separation. *J. Membr. Sci.* **702**, 122758 (2024).

27. L. Wang, Y. Zhou, S. Zha, S. Zhang, J. Jin, An ionic covalent organic framework membrane with confined mobile carriers for stable and efficient carbon dioxide capture. *ACS Sustain. Chem. Eng.* **12**, 18475–18484 (2024).

28. H. Li, S. Zhang, B. Sengupta, H. Li, F. Wang, S. Li, M. Yu, Polystyrene sulfonate (PSS) stabilized polyethylenimine (PEI) membranes fabricated by spray coating for highly effective CO_2/N_2 separation. *J. Membr. Sci.* **657**, 120617 (2022).

29. F. Zhou, H. N. Tien, Q. Dong, W. L. Xu, H. Li, S. Li, M. Yu, Ultrathin, ethylenediamine-functionalized graphene oxide membranes on hollow fibers for CO_2 capture. *J. Membr. Sci.* **573**, 184–191 (2019).

30. Z. Sheng, J. Zhang, J. Liu, Y. Zhang, X. Chen, X. Hou, Liquid-based porous membranes. *Chem. Soc. Rev.* **49**, 7907–7928 (2020).

31. P. Bernardo, E. Drioli, G. Golemme, Membrane gas separation: A review/state of the art. *Ind. Eng. Chem. Res.* **48**, 4638–4663 (2009).

32. N. V. Plechkova, K. R. Seddon, Applications of ionic liquids in the chemical industry. *Chem. Soc. Rev.* **37**, 123–150 (2008).

33. S. Kasahara, E. Kamio, T. Ishigami, H. Matsuyama, Effect of water in ionic liquids on CO_2 permeability in amino acid ionic liquid-based facilitated transport membranes. *J. Membr. Sci.* **415-416**, 168–175 (2012).

34. S. Zhang, H. Li, H. Li, B. Sengupta, S. Zha, S. Li, M. Yu, Negative charge confined amine carriers within the nanowire network for stable and efficient membrane carbon capture. *Adv. Funct. Mater.* **30**, 1–7 (2020).

35. F. Zhou, H. N. Tien, Q. Dong, W. L. Xu, B. Sengupta, S. Zha, J. Jiang, D. Behera, S. Li, M. Yu, Novel carbon-based separation membranes composed of integrated zero- and one-dimensional nanomaterials. *J. Mater. Chem. A* **8**, 1084–1090 (2020).

36. W. Luo, C. Wang, M. Jin, F. Li, H. Li, Z. Zhang, X. Zhang, Y. Liang, G. Huang, T. Zhou, Research progress on nanoconfined ILs in two-dimensional composite membranes for CO_2 capture. *Sep. Purif. Technol.* **330**, 125406 (2024).

37. W. Ying, J. Cai, K. Zhou, D. Chen, Y. Ying, Y. Guo, X. Kong, Z. Xu, X. Peng, Ionic liquid selectively facilitates CO_2 transport through graphene oxide membrane. *ACS Nano* **12**, 5385–5393 (2018).

38. D. Chen, W. Wang, W. Ying, Y. Guo, D. Meng, Y. Yan, R. Yan, X. Peng, CO_2-philic WS_2 laminated membranes with a nanoconfined ionic liquid. *J. Mater. Chem. A* **6**, 16566–16573 (2018).

39. D. Chen, W. Ying, Y. Guo, Y. Ying, X. Peng, Enhanced gas separation through nanoconfined ionic liquid in laminated MoS_2 membrane. *ACS Appl. Mater. Interfaces* **9**, 44251–44257 (2017).

40. C. Shao, W. L. Ong, J. Shiomi, A. J. H. McGaughey, Nanoconfinement between graphene walls suppresses the near-wall diffusion of the ionic liquid [BMIM][PF_6]. *J. Phys. Chem. B* **125**, 4527–4535 (2021).

41. F. Wang, D. K. Behera, K. Friedman, J. Lyu, S. Li, M. Yu, Heterogeneous facilitated transport membrane via ionic liquid-mediated interfacial polymerization for CO_2 separation. *Adv. Funct. Mater.* **35**, 2422445 (2025).

42. K. E. Gutowski, E. J. Maginn, Amine-functionalized task-specific ionic liquids: A mechanistic explanation for the dramatic increase in viscosity upon complexation with CO_2 from molecular simulation. *J. Am. Chem. Soc.* **130**, 14690–14704 (2008).

43. B. Sengupta, Q. Dong, R. Khadka, D. K. Behera, R. Yang, J. Liu, J. Jiang, P. Keblinski, G. Belfort, M. Yu, Carbon-doped metal oxide interfacial nanofilms for ultrafast and precise separation of molecules. *Science* **381**, 1098–1104 (2023).

44. W. R. Bowen, J. S. Welfoot, Modelling of membrane nanofiltration—Pore size distribution effects. *Chem. Eng. Sci.* **57**, 1393–1407 (2002).

45. S. Karan, Z. Jiang, A. G. Livingston, Sub–10 nm polyamide nanofilms with ultrafast solvent transport for molecular separation. *Science* **348**, 1347–1351 (2015).

46. Y. Jin, Q. Song, N. Xie, W. Zheng, J. Wang, J. Zhu, Y. Zhang, Amidoxime-functionalized polymer of intrinsic microporosity (AOPIM-1)-based thin film composite membranes with ultrahigh permeance for organic solvent nanofiltration. *J. Membr. Sci.* **632**, 119375 (2021).

47. Q. Lan, C. Feng, K. Ou, Z. Wang, Y. Wang, T. Liu, Phenolic membranes with tunable sub-10-nm pores for nanofiltration and tight-ultrafiltration. *J. Membr. Sci.* **640**, 119858 (2021).

48. Q. Yang, Y. Su, C. Chi, C. T. Cherian, K. Huang, V. G. Kravets, F. C. Wang, J. C. Zhang, A. Pratt, A. N. Grigorenko, F. Guinea, A. K. Geim, R. R. Nair, Ultrathin graphene-based

membrane with precise molecular sieving and ultrafast solvent permeation. *Nat. Mater.* **16**, 1198–1202 (2017).

49. L. Huang, J. Chen, T. Gao, M. Zhang, Y. Li, L. Dai, L. Qu, G. Shi, Reduced graphene oxide membranes for ultrafast organic solvent nanofiltration. *Adv. Mater.* **28**, 8669–8674 (2016).

50. L. Shen, Q. Shi, S. Zhang, J. Gao, D. C. Cheng, M. Yi, R. Song, L. Wang, J. Jiang, R. Karnik, S. Zhang, Highly porous nanofiber-supported monolayer graphene membranes for ultrafast organic solvent nanofiltration. *Sci. Adv.* **7**, eabg6263 (2021).

51. M. Althuluth, J. P. Overbeek, H. J. van Wees, L. F. Zubeir, W. G. Haije, A. Berrouk, C. J. Peters, M. C. Kroon, Natural gas purification using supported ionic liquid membrane. *J. Membr. Sci.* **484**, 80–86 (2015).

52. Y. Wu, X. Ma, Y. Li, W. Guan, J. Tong, N. Hu, Theoretical and experimental determination of the number of water molecules breaking the structure of a glycine-based ionic liquid. *RSC Adv.* **4**, 10531–10541 (2014).

53. S. Zhao, Z. Zhao, Z. Zha, Z. Jiang, Z. Wang, M. D. Guiver, Amine-rich molecular nodule-assembled membrane having 5 angstrom channels for CO_2/N_2 separation. *Adv. Funct. Mater.* **34**, 1–9 (2024).

54. D. Li, R. Wang, T. S. Chung, Fabrication of lab-scale hollow fiber membrane modules with high packing density. *Sep. Purif. Technol.* **40**, 15–30 (2004).

55. Y. Han, W. Salim, K. K. Chen, D. Wu, W. S. W. Ho, Field trial of spiral-wound facilitated transport membrane module for CO_2 capture from flue gas. *J. Membr. Sci.* **575**, 242–251 (2019).

56. W. Chen, Z. Zhang, C. Yang, J. Liu, H. Shen, K. Yang, Z. Wang, PIM-based mixed-matrix membranes containing MOF-801/ionic liquid nanocomposites for enhanced CO_2 separation performance. *J. Membr. Sci.* **636**, 119581 (2021).

57. Y. Ban, Z. Li, Y. Li, Y. Peng, H. Jin, W. Jiao, A. Guo, P. Wang, Q. Yang, C. Zhong, W. Yang, Confinement of ionic liquids in nanocages: Tailoring the molecular sieving properties of

ZIF-8 for membrane-based CO_2 capture. *Angew. Chem. Int. Ed. Engl.* **54**, 15483–15487 (2015).

58. H. Lin, K. Gong, P. Hykys, D. Chen, W. Ying, Z. Sofer, Y. Yan, Z. Li, X. Peng, Nanoconfined deep eutectic solvent in laminated MXene for efficient CO_2 separation. *Chem. Eng. J.* **405**, 126961 (2021).

59. M. Karunakaran, L. F. Villalobos, M. Kumar, R. Shevate, F. H. Akhtar, K. V. Peinemann, Graphene oxide doped ionic liquid ultrathin composite membranes for efficient CO_2 capture. *J. Mater. Chem. A* **5**, 649–656 (2017).

60. X. Wan, X. Wang, T. Wan, Y. Yan, Z. Ye, X. Peng, Bio-inspired ferromagnetic graphene oxide/magnetic ionic liquid membrane for highly efficient CO_2 separation. *Appl. Mater. Today* **24**, 101164 (2021).

61. W. Guo, S. M. Mahurin, R. R. Unocic, H. Luo, S. Dai, Broadening the gas separation utility of monolayer nanoporous graphene membranes by an ionic liquid gating. *Nano Lett.* **20**, 7995–8000 (2020).

62. J. Albo, T. Tsuru, Thin ionic liquid membranes based on inorganic supports with different pore sizes. *Ind. Eng. Chem. Res.* **53**, 8045–8056 (2014).

63. H. Li, F. Wang, H. Li, B. Sengupta, D. K. Behera, S. Li, M. Yu, Ultra-selective membrane composed of charge-stabilized fixed carrier and amino acid-based ionic liquid mobile carrier for highly efficient carbon capture. *Chem. Eng. J.* **453**, 139780 (2023).

64. X. M. Zhang, Z. H. Tu, H. Li, L. Li, Y. T. Wu, X. B. Hu, Supported protic-ionic-liquid membranes with facilitated transport mechanism for the selective separation of CO_2. *J. Membr. Sci.* **527**, 60–67 (2017).

65. D. K. Behera, F. Wang, B. Sengupta, K. Friedman, S. Li, M. Yu, A facilitated transport membrane composed of amine-containing ionic liquid confined in a GO/CNT network for highly efficient carbon capture. *J. Membr. Sci.* **712**, 123177 (2024).

66. S. M. Mahurin, J. S. Lee, G. A. Baker, H. Luo, S. Dai, Performance of nitrile-containing anions in task-specific ionic liquids for improved CO_2/N_2 separation. *J. Membr. Sci.* **353**, 177–183 (2010).

67. M. Y. Abdelrahim, C. F. Martins, L. A. Neves, C. Capasso, C. T. Supuran, I. M. Coelhoso, J. G. Crespo, M. Barboiu, Supported ionic liquid membranes immobilized with carbonic anhydrases for CO_2 transport at high temperatures. *J. Membr. Sci.* **528**, 225–230 (2017).

68. E. Santos, J. Albo, A. Irabien, Acetate based supported ionic liquid membranes (SILMs) for CO_2 separation: Influence of the temperature. *J. Membr. Sci.* **452**, 277–283 (2014).

69. J. Yin, C. Zhang, Y. Yu, T. Hao, H. Wang, X. Ding, J. Meng, Tuning the microstructure of crosslinked Poly(ionic liquid) membranes and gels via a multicomponent reaction for improved CO_2 capture performance. *J. Membr. Sci.* **593**, 117405 (2020).

70. J. Zhou, M. M. Mok, M. G. Cowan, W. M. McDanel, T. K. Carlisle, D. L. Gin, R. D. Noble, High-permeance room-temperature ionic-liquid-based membranes for CO_2/N_2 separation. *Ind. Eng. Chem. Res.* **53**, 20064–20067 (2014).

71. J. Y. Lim, J. K. Kim, C. S. Lee, J. M. Lee, J. H. Kim, Hybrid membranes of nanostructrual copolymer and ionic liquid for carbon dioxide capture. *Chem. Eng. J.* **322**, 254–262 (2017).

72. S. Janakiram, L. Ansaloni, S. A. Jin, X. Yu, Z. Dai, R. J. Spontak, L. Deng, Humidity-responsive molecular gate-opening mechanism for gas separation in ultraselective nanocellulose/IL hybrid membranes. *Green Chem.* **22**, 3546–3557 (2020).

73. V. A. Kusuma, M. K. Macala, J. Liu, A. M. Marti, R. J. Hirsch, L. J. Hill, D. Hopkinson, Ionic liquid compatibility in polyethylene oxide/siloxane ion gel membranes. *J. Membr. Sci.* **545**, 292–300 (2018).

74. M. Sandru, T. J. Kim, W. Capala, M. Huijbers, M. B. Hägg, Pilot scale testing of polymeric membranes for CO_2 capture from coal fired power plants. *Energy Procedia* **37**, 6473–6480 (2013).

75. X. Xu, J. Dong, X. Xiao, X. Zhao, Q. Zhang, Constructing thin and cross-linked polyimide membranes by interfacial reaction for efficient CO_2 separation. *ACS Sustain. Chem. Eng.* **9**, 5546–5556 (2021).

76. J. M. P. Scofield, P. A. Gurr, J. Kim, Q. Fu, S. E. Kentish, G. G. Qiao, Development of novel fluorinated additives for high performance CO_2 separation thin-film composite membranes. *J. Membr. Sci.* **499**, 191–200 (2016).

77. L. S. White, K. D. Amo, T. Wu, T. C. Merkel, Extended field trials of Polaris sweep modules for carbon capture. *J. Membr. Sci.* **542**, 217–225 (2017).

78. P. Li, Z. Wang, W. Li, Y. Liu, J. Wang, S. Wang, High-performance multilayer composite membranes with mussel-inspired polydopamine as a versatile molecular bridge for CO_2 separation. *ACS Appl. Mater. Interfaces* **7**, 15481–15493 (2015).

79. S. Li, Z. Wang, X. Yu, J. Wang, S. Wang, High-performance membranes with multi-permselectivity for CO_2 separation. *Adv. Mater.* **24**, 3196–3200 (2012).

80. K. J. Howe, M. M. Clark, Fouling of microfiltration and ultrafiltration membranes by natural waters. *Environ. Sci. Technol.* **36**, 3571–3576 (2002).
